# Compressed and Expanded Lattices - Barriers to Spin-State Switching in Mn^3+^ Complexes

**DOI:** 10.1021/acs.cgd.2c01284

**Published:** 2023-05-24

**Authors:** Michelle M. Harris, Irina A. Kühne, Conor T. Kelly, Vibe B. Jakobsen, Ross Jordan, Luke O’Brien, Helge Müller-Bunz, Solveig Felton, Grace G. Morgan

**Affiliations:** † School of Chemistry, University College Dublin, Belfield, Dublin, D04 V1W8, Ireland; ‡ Department of Functional Materials, FZU - Institute of Physics - Czech Academy of Sciences, Na Slovance 1999/2, Prague 8, 182 21, Czech Republic; § Centre for Quantum Materials and Technologies, School of Mathematics and Physics, Queen’s University Belfast, Belfast BT7 1NN, United Kingdom

## Abstract

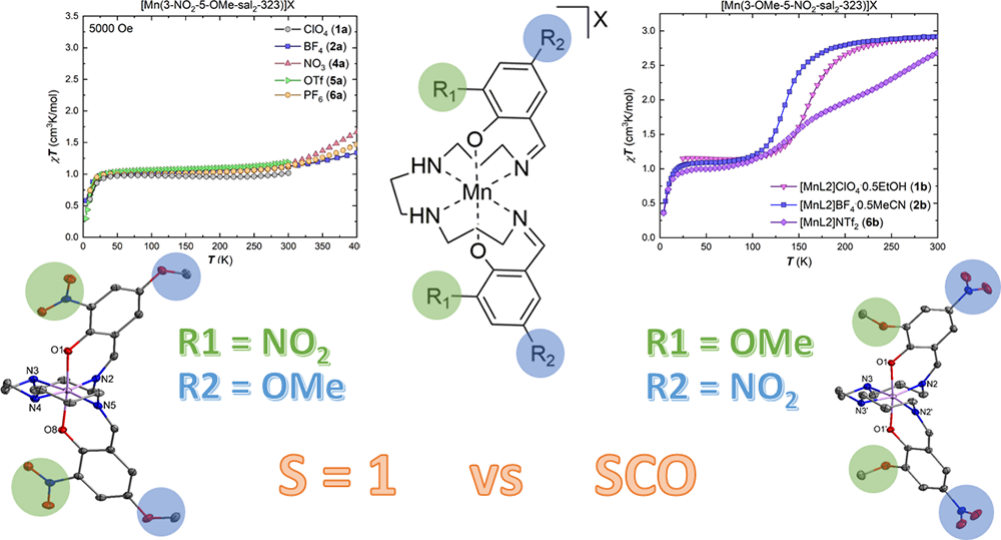

We report the structural and magnetic properties of two new Mn^3+^ complex cations in the spin crossover (SCO) [Mn(R-sal_2_323)]^+^ series, in lattices with seven different counterions in each case. We investigate the effect on the Mn^3+^ spin state of appending electron-withdrawing and electron-donating groups on the phenolate donors of the ligand. This was achieved by substitution of the *ortho* and *para* positions on the phenolate donors with nitro and methoxy substituents in both possible geometric isomeric forms. Using this design paradigm, the [MnL1]^+^ (**a**) and [MnL2]^+^ (**b**) complex cations were prepared by complexation of Mn^3+^ to the hexadentate Schiff base ligands with 3-nitro-5-methoxy-phenolate or 3-methoxy-5-nitro-phenolate substituents, respectively. A clear trend emerges with adoption of the spin triplet form in complexes **1a**–**7a,** with the 3-nitro-5-methoxy-phenolate donors, and spin triplet, spin quintet and thermal SCO in complexes **1b**–**7b** with the 3-methoxy-5-nitro-phenolate ligand isomer. The outcomes are discussed in terms of geometric and steric factors in the 14 new compounds and by a wider analysis of electronic choices of Mn^3+^ with related ligands by comparison of bond length and angular distortion data of previously reported analogues in the [Mn(R-sal_2_323)]^+^ family. The structural and magnetic data published to date suggest a barrier to switching may exist for high spin forms of Mn^3+^ in those complexes with the longest bond lengths and highest distortion parameters. A barrier to switching from low spin to high spin is less clear but may operate in the seven [Mn(3-NO_2_-5-OMe-sal_2_323)]^+^ complexes **1a**–**7a** reported here which were all low spin in the solid state at room temperature.

## Abstract 2

Structural and magnetic data of 15 new manganese(III) complexes from the [Mn(R-sal_2_-323)]^+^ series are compared with more than 200 published examples in order to analyze the correlation between distortion parameters and spin state and to determine if barriers to spin-state switching from either the low spin or high spin states arise in complexes with very large or very small distortion.

## Introduction

Thermal spin crossover (SCO) in transition-metal complexes is one of the most intensively studied types of molecular switching, and the phenomenon has been realized in a variety of material types.^1−3^ These include single crystals of various dimensions,^4^ polymers and composites,^5^ discrete nano-objects,^6^ thin and thick films,^7^ liquid crystals,^8,9^ micellar assemblies^10,11^ and ionic liquids.^12^ Conserving the phenomenon beyond the solid state assumes that the switching is indeed at the level of the single molecule rather than a consequence of the ensemble. In this respect, it is noteworthy that examples of spin-state switching in solution or other soft media have only been reported in Fe^2+^, Fe^3+^ or Co^2+^ complexes.^9^ In contrast, emerging data on thermal spin-state switching in Mn^3+^ suggests that the effect may only be realized in the solid state and that there is a critical distortion value above which switching from high spin (HS) to low spin (LS) is energetically disfavored.^13^

**Scheme 1:**
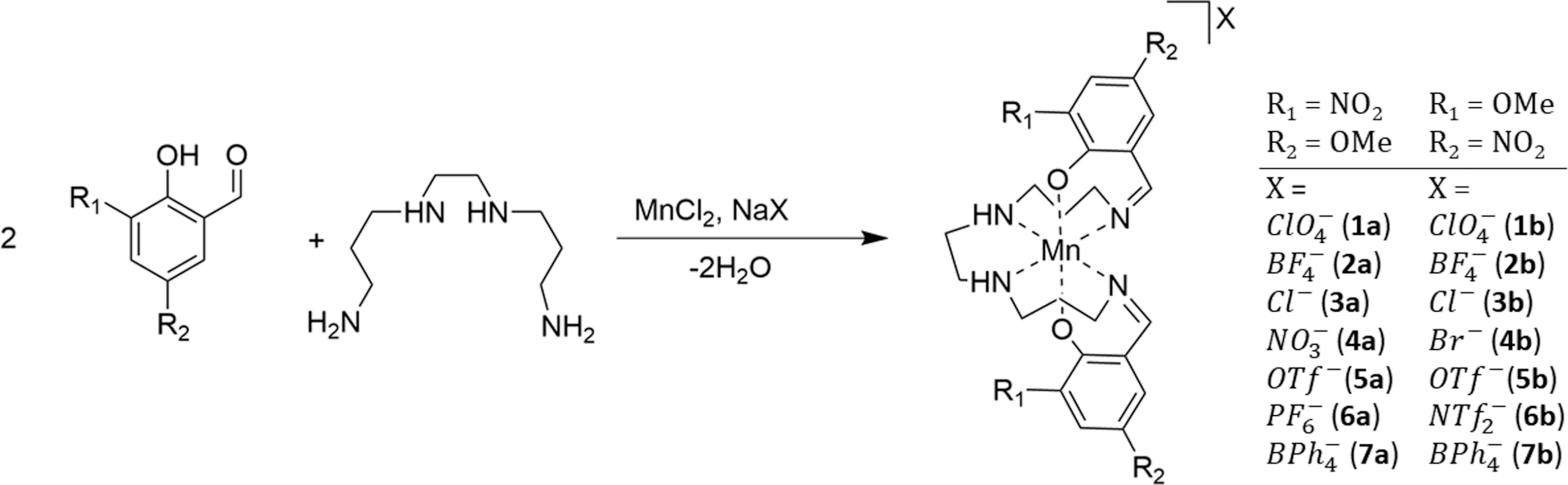
Synthesis of [Mn(3-NO_2_-5-OMe-sal_2_-323)]X Complexes **1a**–**7a** and [Mn(3-OMe-5-NO_2_-sal_2_-323)]X complexes **1b**–**7b**

While the prevalence of thermal spin-state switching in Mn^3+^ complexes has been examined with a few ligand types,^14−17^ the coordinatively elastic R-sal_2_323 generic Schiff base (see Scheme 1 for an example) is the most widely used ligand to promote SCO with this ion,^18^ and manganese complexes of this type are well suited to crystal engineering.^19,20^ These routine crystal engineering studies have demonstrated that intermolecular interactions and subtle lattice pressures are particularly important in modulating the distance between spin labile sites in manganese complexes. Filling the space between spin labile Mn^3+^ sites with sterically demanding ligand substituents may constrain the spin state to the triplet form, for example, by replacing phenolate donors with larger naphthol analogues.^21^ Similarly the position of any R substituent on the phenolate donor is important in modulating the spin state choice. We have recently reported the spin state choices of [Mn(3-NO_2_-sal_2_323)]^+^ and [Mn(5-NO_2_-sal_2_323)]^+^ complexes in lattices with different counterions which revealed a slight preference for the spin triplet form when the nitro group is *ortho* and for the spin quintet form when *para* to the oxygen donor.^22^ In this case, DFT showed that the ligand field energies generated by the 3-NO_2_-sal_2_323 and 5-NO_2_-sal_2_323 ligands were comparable and the preference for the triplet or quintet forms of Mn^3+^ was therefore ascribed to crystal packing. In other examples, we have noted a tendency for stabilization of HS Mn^3+^ in BPh_4_^–^ salts, possibly due to the absence of counterion-mediated hydrogen bonding to mediate the spin transition.^20,22,23^ Perhaps most striking is the effect of appending long alkyl groups onto the ligands in [Mn(R-sal_2_323)]^+^ complexes. In comparable Fe^3+^ complexes, this strategy was used to great effect to prepare SCO amphiphiles in a variety of material forms including Langmuir films at the air–water interface,^24^ micelles in organic solvents,^11^ and template assembly of 1D nanowires.^6^ In these examples with Fe^3+^, the SCO function was retained in all cases, often with a modified thermal evolution profile. In contrast, using the alkylation approach with [Mn(R-sal_2_323)]^+^ complexes typically switched off SCO in the solid state and the HS manganese complexes which were recovered had some of the longest reported bond lengths despite the ability of the nonalkylated analogues to stabilize different spin states over a thermal gradient.^13^

In the present work, we report the structural and magnetic properties of two new [Mn(R-sal_2_323)]^+^ complex cations in lattices with seven different counterions in each case (Scheme 1). The complex cations are distinguished by the choice of isomeric Schiff base ligand with 3-nitro-5-methoxy-phenolate (L1) or 3-methoxy-5-nitro-phenolate (L2) donors, respectively. Thus, both ligands host both electron-donating and electron-withdrawing groups, and a clear preference for spin triplet Mn^3+^ emerges for the complexes with the 3-nitro-5-methoxy-phenolate donors. While it is tempting to ascribe this to the electronic nature of the phenolate donor substituent, an alternative explanation is that the difference is due to packing. This is indicated by modification of the packing in the BPh_4_^–^ salt of the complex with 3-nitro-5-methoxy-phenolate donors, which irreversibly switches from LS to HS when lattice solvent is removed. In this work, we examine the factors which may contribute to spin state choice in such [Mn(R-sal_2_323)]^+^ systems and how the determining factors may be distinguished.

## Results and Discussion

### Synthetic Procedure

The condensation reaction of 3-nitro-5-methoxysalicylaldehyde and 3-methoxy-5-nitrosalicylaldehyde respectively in a 2:1 ratio with 1,2-bis(3-aminopropylamino)ethane led to the formation of two hexadentate Schiff base ligands (Scheme 1). The reaction of a Mn^2+^ salt with these ligands led to an aerial oxidation and the crystallization of two families of Mn^3+^ mononuclear compounds in the form of dark red/black crystals or microcrystalline powders. The perchlorate, nitrate, chloride and bromide complexes were directly synthesized from the respective manganese(II) salt, while the remaining complexes were formed using salt metathesis.

The structures of were established by single crystal X-ray diffraction in most cases, and the bulk samples of all were characterized using elemental analysis and SQUID magnetometry. In several cases the complexes crystallized with solvation, the full formula is shown for each of the single crystal samples in the crystallographic tables in the Supporting Information, Tables A1–B3. The identity and percentage of solvent molecules determined by single crystal diffraction did not match that of the bulk in all cases, and the degree of solvation in the bulk samples used for SQUID magnetometry was estimated from the elemental analysis results (measured for C, H and N). The full formulae used for magnetic measurements are given in the Experimental Section.

### Magnetic Properties

**Figure 1:**
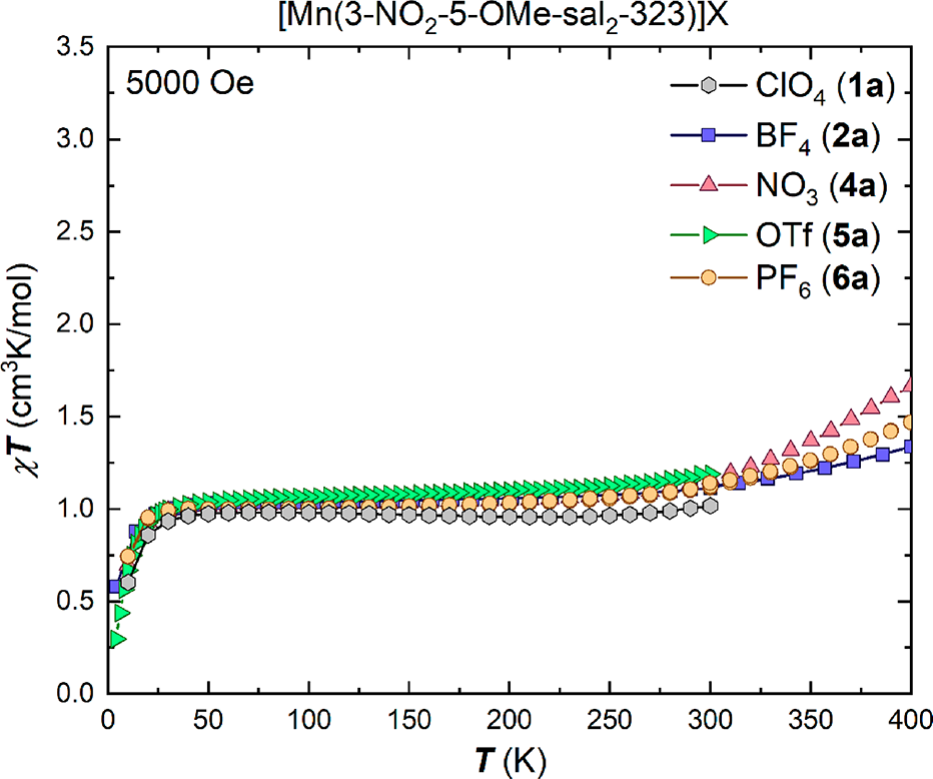
Plots of χ_M_*T* versus *T* for the complexes of the [Mn(3-NO_2_-5-OMe-sal_2_-323)]X family, **1a**, **2a** and **4a**–**6a** in the temperature range 5–400 K in cooling mode using an applied dc field of 5000 Oe.

**Figure 2:**
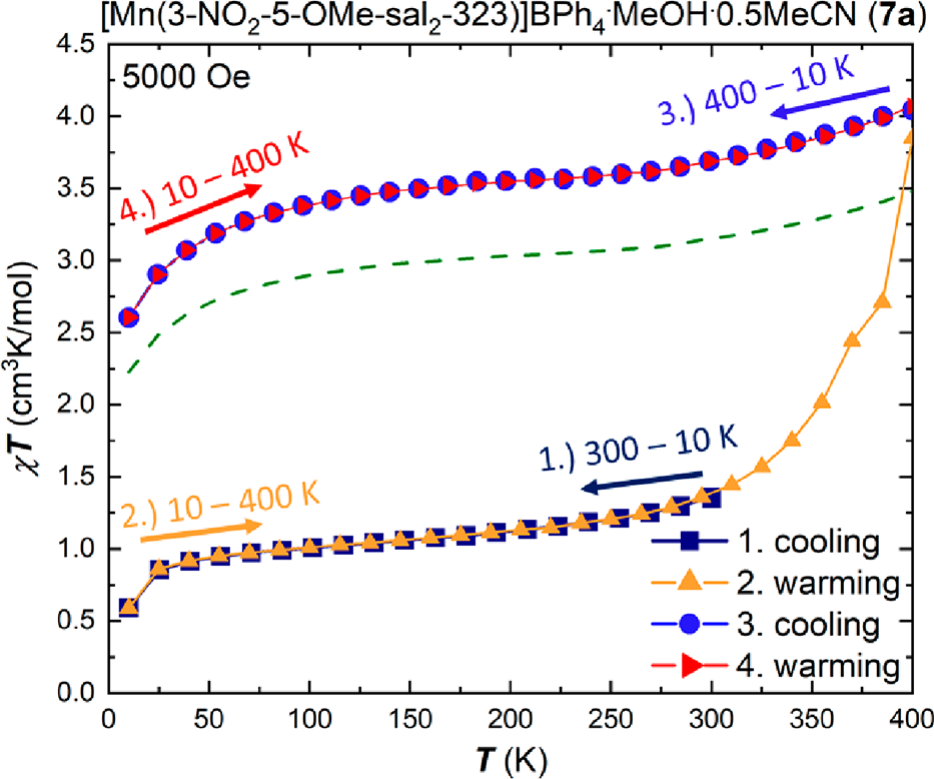
Temperature dependent χ_M_*T* plot for [Mn(3-NO_2_-5-OMe-sal_2_-323)]BPh_4_·MeOH·0.5MeCN, **7a·MeOH·0.5MeCN**, in the temperature range 5–400 K in two cooling-warming cycles, using an applied dc field of 5000 Oe (adjusted molecular weight after solvent loss, green dashed line).

**Figure 3:**
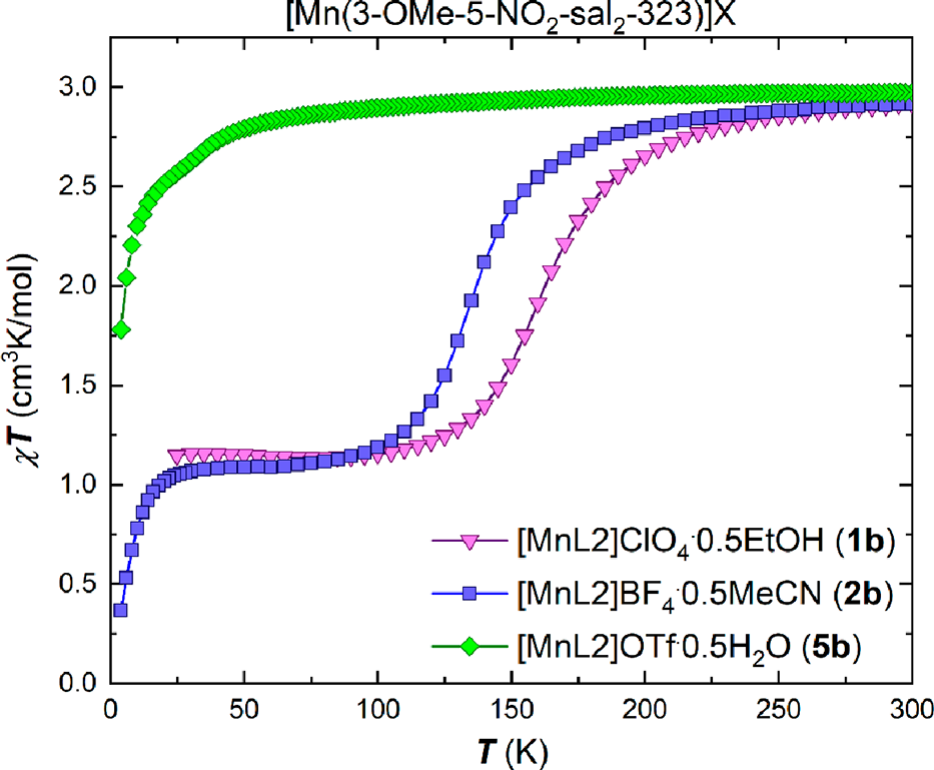
Plots of χ_M_*T* versus *T* for the complexes of the [Mn(3-OMe-5-NO_2_-sal_2_-323)]X family, **1b**, **2b** and **5b** in the temperature range 5–300 K in cooling mode using an applied dc field of 5000 Oe for **1b** and **2b** and 1000 Oe for **5b**.

**Figure 4:**
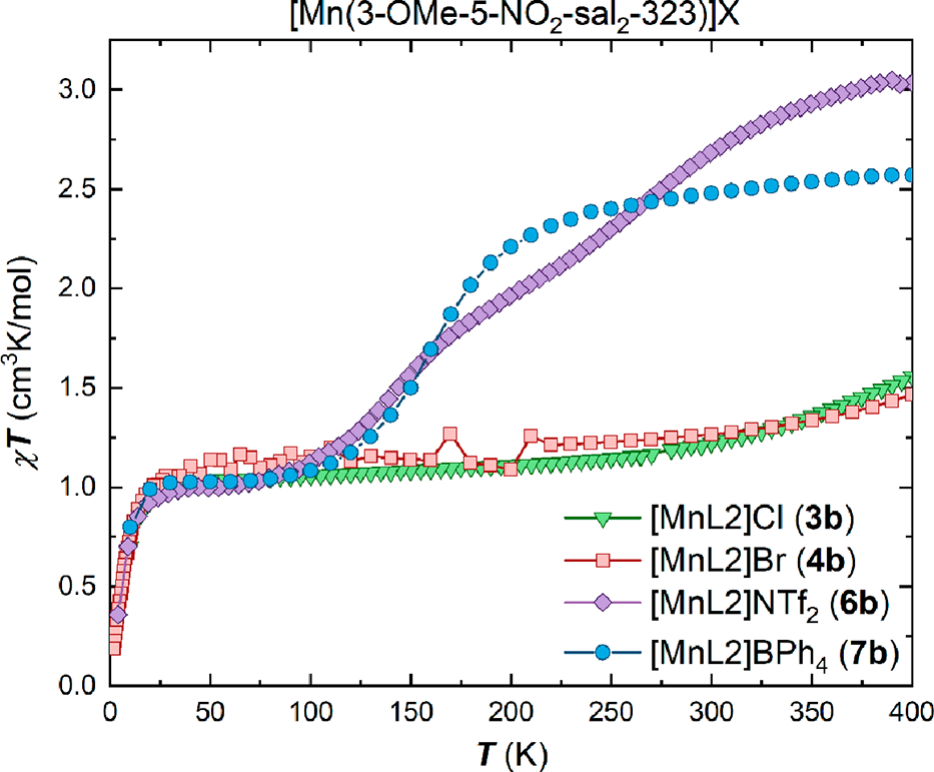
Plots of χ_M_*T* versus *T* for the complexes of the [Mn(3-OMe-5-NO_2_-sal_2_-323)]X family, **3b**, **4b**, **6b** and **7b** in the temperature range 5–400 K in cooling mode using an applied dc field of 5000 Oe for **3b** and **7b** and 1000 Oe for **4b** and **6b**.

Magnetic susceptibility data of the bulk samples of compounds (**1**–**7**) in both the (**a**) and (**b**) series were collected via SQUID magnetometry in cooling and heating modes in an applied dc field of 5000 Oe in the temperature range 5–300 K, and up to 400 K in some cases, as shown in the χ_M_*T* versus *T* plots in Figures 1–4. There is no evidence of reversible thermal hysteresis for any of the samples. The new thermal pathway followed by [Mn(3-NO_2_-5-OMe-sal_2_-323)]BPh_4_·MeOH·0.5MeCN, **7a·MeOH·0.5MeCN**, after one heating cycle to 400 K is ascribed to solvent loss *vide infra*, and there is no thermal hysteresis in the spin-state switching of the desolvated complex.

The magnetic properties for five of the [Mn(3-NO_2_-5-OMe-sal_2_-323)]X complexes, series (**a**), are shown in Figure 1. For compounds, **1a**, **2a** and **4a**–**6a**, the χ_M_*T* versus *T* plots clearly highlight the preference for the spin-triplet *S* = 1 state for temperatures up to 300 K (χ_M_*T* = 1.0 cm^3^K/mol for *S* = 1 using g = 2). The small increase in the χ_M_*T* product in those complexes which were heated to 400 K indicates that thermal SCO is achievable within this coordination sphere.

The magnetic properties of the solvated BPh_4_^–^ salt **7a**·MeOH·0.5MeCN were investigated on a freshly filtered sample to avoid solvent loss, and the magnetic susceptibility data are shown as χ_M_*T* versus *T* plots in Figure 2. The initial χ_M_*T* product was measured in cooling mode from 300 to 10 K (blue squares, Figure 2) and clearly highlights that the sample is in the spin-triplet *S* = 1 state over the measured temperature range. Upon warming back to room temperature from 5 K (orange triangles, Figure 2), the χ_M_*T* product follows the same slope up to 300 K, but on further increase in temperature to 400 K, the χ_M_*T* product steadily increases reaching a value of 3.8 cm^3^K/mol. In order to check for SCO behavior, the susceptibility was measured in an additional step in cooling mode (blue circles, Figure 2). In this cooling mode, the χ_M_*T* product is almost constant at a value of 3.5 cm^3^K/mol in the measured temperature range of 300–80 K. The drop at lower temperatures is assigned to ZFS rather than a spin transition.

Upon a final cycle of heating (red triangles, Figure 2), the χ_M_*T* product follows the slope of the most recent previous cycle (blue circles). This suggests loss of the methanol and acetonitrile solvents in the crystal lattice, which is followed by a change in the spin state. The slightly higher χ_M_*T* value of 3.5 cm^3^K/mol (expected value of 3.0 cm^3^K/mol for *S* = 2 with *g* = 2) can be explained by the changing molar mass due to the solvent loss. This decreases the molecular weight by 5.5%, and after adjusting the molecular weight to account for the solvent loss, the χ_M_*T* product fits well the theoretical value of an *S* = 2 system (green dashed line, Figure 2).

The magnetic properties for the [Mn(3-OMe-5-NO_2_-sal_2_-323)]X complexes, series (**b**), are shown in Figures 3 and 4. For the solvated compounds, **1b** and **2b**, the χ_M_*T* versus *T* plots (Figure 3) show spin transition with similar profiles, as expected for isostructural compounds. The χ_M_*T* data reveal that the compounds are in the spin-triplet *S* = 1 state at low temperatures before transition to the spin quintet *S* = 2 form at temperatures beyond 200 K.

The *T*_1/2_ values were found to be 165 K for **1b**·0.5EtOH and 135 K for **2b**·0.5MeCN highlighting the effect of the smaller BF_4_^–^ anion on the transition temperature. The triflate containing complex **5b**·0.5H_2_O remains in the spin quintet, *S* = 2, form with a value of χ_M_*T* = 2.97 cm^3^K/mol over the entire measured temperature range.

Compounds **3b** and **4b**, containing the small halide counterions (Figure 4), show a clear preference for the spin-triplet *S* = 1 state over the whole measured temperature range up to 400 K. Only a small increase in the χ_M_*T* product can be observed at temperatures beyond 300 K, leading to a small fraction of the spin quintet form. The values of 1.56 cm^3^K/mol for **3b** and 1.47 cm^3^K/mol for **4b** at 400 K indicate a 28% spin quintet fraction for **3b** and 24% fraction for **4b**, respectively.

The magnetic plot of **6b** (Figure 4) reveals a broad stepped spin transition profile in a temperature window between 100 and 400 K with a plateau at the *T*_1/2_ value of 2.0 cm^3^K/mol at 206 K. The χ_M_T product exhibits a value of 1.00 cm^3^K/mol up to 110 K, the pure spin triplet form, while the value of 3.00 cm^3^K/mol reached at temperatures around 400 K indicates a complete transition to an *S* = 2 system. The minimum in the first derivative of the χ_M_*T* product of complex **6b** (Figure S1) highlights the existence of a plateau at 209 K in the χ_M_*T* product, which is surrounded by two turning points at 139 and 269 K, respectively. Stepped spin transitions typically signal a spin-state ordered phase, with a 1:1 or some other ratio of spin states and the concomitant appearance of crystallographically nonequivalent metal centers in the asymmetric unit.^25^ A spin-state ordered phase was not, however, detected for compound **6b** in our experimental setup.

Complex **7b** containing the large tetraphenylborate counterion exhibits an incomplete spin transition profile (Figure 4) in the measured temperature range up to 400 K. At temperatures below 120 K, **7b** remains in the *S* = 1 triplet state. Upon increase in temperature, the χ_M_*T* product follows a steep increase up to 220 K where χ_M_*T* reaches a value of 2.31 cm^3^K/mol, before following a more gradual increase without reaching a value of 3.0 cm^3^K/mol which would be indicative of a complete transition to a full HS compound. The value of 2.47 cm^3^K/mol indicates a 70% fraction of one *S* = 2 component. Two independent samples were prepared and measured, and both show the same incomplete SCO behavior (Figure S2).

### Structural Details

#### Crystal Structures of [Mn(3-NO_2_-5-OMe-sal_2_-323)]X, Series **a**

**Figure 5:**
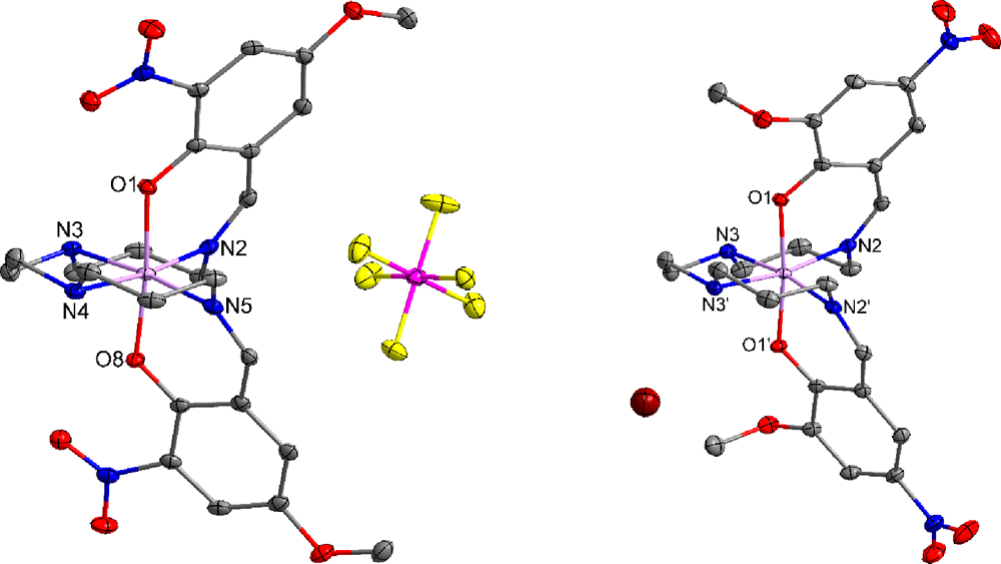
Molecular structure of [Mn(3-NO_2_-5-OMe-sal_2_-323)]PF_6_ (**6a**) (left) and [Mn(3-OMe-5-NO_2_-sal_2_-323)]Br (**4b**) (right) measured at 100 K shown as an example for each series of compounds (hydrogen atoms omitted for clarity).

The structures of the [Mn(3-NO_2_-5-OMe-sal_2_323)]X type compounds, series (**a**), were determined at 100 K for the chloride, **3a**, nitrate, **4a**, hexafluorophosphate, **6a**, and tetraphenylborate **7a**, anion-containing complexes. In all structures, the hexadentate Schiff base ligand, 3-NO_2_-5-OMe-sal_2_-323, chelates the central Mn^3+^ ion in a *pseudo*-octahedral fashion with two *trans*-phenolate donors, two *cis*-amine and two *cis*-imine donor atoms, in the same way as previously observed for manganese(III) complexes with this type of ligand. The structure of compound **6a** is shown in Figure 5 as a representative example of the coordination sphere in compound series (**a**).

Complex **3a** crystallizes in the monoclinic space group *P*2/*c* with one unique [Mn(3-NO_2_-5-OMe-sal_2_-323)]^+^ cation and one chloride anion. The nitrate containing complex **4a** was found to cocrystallize in two different forms. On the one hand, **4a** formed block-shaped crystals which were found to crystallize in the monoclinic space group *C*2/*c*, and on the other hand, there are needle-shaped crystals which were found in the hexagonal space group *P*6_5_22. Both lattices contain one-half-occupancy Mn^3+^ cationic species and one full anion. Complex **6a**, containing the octahedral hexafluorophosphate anion, crystallizes in the triclinic space group *P*1̅ with one full occupancy cation and anion, respectively.

While compounds **3a**, **4a** and **6a** crystallize solvent free, complex **7a** with the tetraphenylborate anion was found to crystallize with either one methanol together with half of an acetonitrile molecule or two ethanol molecules in the crystal lattice yielding **7a·MeOH·0.5MeCN** and **7a·2EtOH**. Both solvatomorphs crystallize isostructurally in the same monoclinic space group *P*2_1_/*n* containing one independent [Mn(3-NO_2_-5-OMe-sal_2_-323)]^+^ cation and one BPh_4_^–^ anion. The magnetic properties were measured on **7a·MeOH·0.5MeCN**.

Selected crystallographic data and structure refinements are summarized in Tables S-A1 and S-A2, in the Supporting Information.

#### Crystal Structures of [Mn(3-OMe-5-NO_2_-sal_2_-323)]X, Series **b**

The structures of the [Mn(3-OMe-5-NO_2_-sal_2_-323)]X type compounds, series (**b**), were determined for **1b·0.5EtOH**, **2b·0.5MeCN**, **3b**, **4b**, **5b·0.5H**_**2**_**O**, **6b** and **7b** at 100 K, and at an additional high temperature for **4b** (293 K) and **6b** (180 K). Selected crystallographic data and structure refinements are summarized in Tables S-B1–B3, Supporting Information.

Similar to the complexes in the isomeric series (**a**), the hexadentate Schiff base ligand, 3-OMe-5-NO_2_-sal_2_-323, chelates the central Mn^3+^ ion in a *pseudo*-octahedral fashion with two *trans*-phenolate donors, two *cis*-amine and two *cis*-imine donor atoms. The structure of compound **4b** is shown in Figure 5 as a representative example of the coordination sphere in compound series (**b**).

Complexes **1b·0.5EtOH** and **2b·0.5MeCN**, containing the tetrahedral perchlorate and tetrafluoroborate anions, are isostructural and crystallize in the monoclinic space group *C*2/*c* with *Z* = 4. The asymmetric unit contains one full-occupancy [Mn(3-OMe-5-NO_2_-sal_2_-323)]^+^ and one full-occupancy anion. In addition, there is half of one solvent molecule per asymmetric unit. In the case of complex **1b·0.5EtOH**, this is comprised of half of an ethanol molecule, while in complex **2b·0.5MeCN**, this position is filled by half of an acetonitrile molecule.

The halide containing complexes, **3b** and **4b**, crystallize in the polar and orthorhombic space group *Pba*2 with *Z* = 2. The structure of complex **4b** was determined at 100 and 293 K without any significant change in the unit cell parameters upon heating. The asymmetric unit contains in both cases one full-occupancy cation and one full-occupancy halide anion.

The hydrated complex **5b·0.5H**_**2**_**O** crystallizes in the monoclinic space group *P*2_1_/*c* with *Z* = 4. The asymmetric unit of **5b·0.5H**_**2**_**O** contains two full-occupancy Mn^3+^ complex cations, two full-occupancy triflate anions and one molecule of water.

The structure of **6b** was determined at 100 and 180 K, while attempts to obtain a higher temperature structure were unsuccessful. At both temperatures, **6b** crystallizes in the triclinic space group *P*1̅ with *Z* = 4. The asymmetric unit contains two full-occupancy [Mn(3-OMe-5-NO_2_-sal_2_-323)]^+^ cations and two bistriflimide anions. At 100 K, one of the bistriflimide counterions shows a disorder of the CF_3_ moiety as well as the SO_2_ part of one-half of the anion with a site occupation ratio of 1:4. Upon increase in temperature to 180 K, the same bistriflimide anion now exhibits disorder at both ends of the molecule with similar site occupation factors.

Complex **7b**, containing the large tetraphenylborate anion, crystallizes in the monoclinic space group *P*2_1_/*c* with *Z* = 4. The asymmetric unit consists of one complex cation and one full anion.

### Bond Length Changes

In Mn^3+^ SCO compounds of the [Mn(R-sal_2_323)]^+^ type, the equatorial nitrogen bond lengths show a visible increase upon spin transition, while the axial oxygen bond distances stay almost constant. The Mn–N_imine_ bond lengths are typically 1.95–2.00 Å in the spin triplet form (*S* = 1), increasing to 2.05–2.18 Å within the spin quintet form (*S* = 2), while the Mn–N_amine_ bond lengths change from 2.03–2.10 Å to 2.18–2.30 Å upon spin transition. The bond lengths for the complexeare given in Table 1, series (a) and for series (**b**) in Tables 2 and 3.

**Table 1:** Bond Lengths of [Mn(3-NO_2_-5-OMe-sal_2_-323)]X Complexes **3a**, **4a**, **6a** and **7a**

Mn–X	Cl^–^ (**3a**)	NO_3_^–^ (**4a**)		PF_6_^–^ (**6a**)	BPh_4_^–^ (**7a**)	
Temp (K)	100	100		100	100	
Polymorph^a^		a	b		c	d
Mn–O_phen_	1.891	1.879	1.884	1.885	1.872	1.873
				1.876	1.872	1.885
Mn–N_imine_	1.990	1.987	1.970	1.969	1.977	1.980
				1.970	1.982	1.983
Mn–N_amine_	2.049	2.049	2.050	2.044	2.040	2.042
				2.048	2.047	2.049
spin state	*S* = 1	*S* = 1		*S* = 1	S_1_ = 1	S_2_ = 1

a Polymorph: a = mor780 (needle; *P*6_5_22); b = mor781 (block; *C*2/*c*); c = mor706:7a·MeOH·0.5MeCN; and d = mor771:7a·2EtOH.

**Table 2:** Bond Lengths of the [Mn(3-OMe-5-NO_2_-sal_2_-323)]X Complexes **1b**–**4b**

Mn–X	**ClO_4_^–^** (**1b**)	**BF_4_^–^** (**2b**)	**Cl^–^** (**3b**)	**Br^–^** (**4b**)	
Temp (K)	100	100	100	100	293
Mn–O_phen_	1.868	1.867	1.874	1.877	1.876
	1.875	1.878			
Mn–N_imine_	1.995	1.991	1.991	1.988	1.990
	2.003	1.995			
Mn–N_amine_	2.064	2.058	2.035	2.028	2.033
	2.069	2.062			
spin state	*S* = 1	*S* = 1	*S* = 1	*S* = 1	*S* = 1

**Table 3:** Bond Lengths of the [Mn(3-OMe-5-NO_2_-sal_2_-323)]X Complexes **5b**–**7b**

Mn–X	OTf^**–**^ (**5b**)	NTf_2_^–^ (**6b**)	NTf_2_^–^ (**6b**)	BPh_4_^–^ (**7b**)
Temp (K)	100	100	180	100
Mn–O_phen_	1.876; 1.868	1.868; 1.871	1.868; 1.863	1.864
	1.881; 1.876	1.884; 1.879	1.883; 1.879	1.885
Mn–N_imine_	2.053; 2.114	1.989; 1.994	1.995; 2.038	2.005
	2.136; 2.130	1.989; 2.008	1.995; 2.121	2.005
Mn–N_amine_	2.155; 2.223	2.045; 2.070	2.052; 2.149	2.058
	2.267; 2.223	2.052; 2.090	2.056; 2.224	2.062
spin state	*S*_1_ = mixed; *S*_2_ = 2	*S*_1_ = 1; *S*_2_ = 1	*S*_1_ = 1; *S*_2_ = IS	*S* = 1

The observed bond lengths around the Mn^3+^ center of complexes **3a**, the two polymorphs of **4a** and **6a**, all exhibit short Mn–N_amine_ and Mn–N_imine_ distances with all imine bond lengths smaller than 2.0 Å and the amine bond lengths in the range between 2.04–2.05 Å (see Table 1). These are indicative of Mn^3+^ in the triplet spin state and are in good agreement with the observed magnetic properties.

The structure of **7a·MeOH·0.5MeCN** at 100 K exhibits two independent Mn^3+^ sites which both show Mn–N_amine_ and Mn–N_imine_ bond lengths that are indicative of *S* = 1 (see Table 1), which fits to the magnetic properties before solvent loss.

The bond lengths of compounds **1b**·0.5EtOH and **2b**·0.5MeCN at 100 K are compared in Table 2. The short Mn–N_amine_ and Mn–N_imine_ distances indicate that both complexes are in the spin triplet form at 100 K, which is in good agreement with the magnetic properties (Figure 3). The two complexes containing halide anions, **3b** and **4b**, were found to remain in the spin triplet form up to 400 K (Figure 4). This is supported by the short bond lengths found within **3b** and **4b** (Table 2). The structure of **4b** was determined at 100 and 293 K, and both sets of bond lengths are indicative of a Mn^3+^ center in the triplet spin state.

Even though the magnetic properties of the triflate containing complex **5b**·0.5H_2_O indicate the spin quintet, *S* = 2, form (Figure 3), the structure determined at 100 K contains two independent Mn^3+^ sites which exhibit different bond lengths (Table 3). While Mn2 shows long Mn–N_amine_ and Mn–N_imine_ bond lengths which are clearly indicative of the spin quintet form, the bond lengths around Mn1 show more distortion with each one short and one long Mn–N_amine_ and Mn–N_imine_ bond.

The structure of the bistriflimide complex **6b** was determined at 100 and 180 K, and the bond lengths are summarized in Table 3. At both measured temperatures, the structure contains two independent Mn^3+^ sites. At 100 K, both sites exhibit bond lengths that are indicative of the spin triplet form with short Mn–N_amine_ and Mn–N_imine_ bond lengths. Upon increase in temperature to 180 K, the complex around Mn1 retains short bond lengths, while those around Mn2 partially increase but do not attain the expected values for a spin triplet Mn^3+^ by 180 K. The increase of the Mn–N_amine_ and Mn–N_imine_ bond lengths is similarly distorted as observed within the Mn1 site of complex **5b**, with each containing one short and one long amine and imine distance.

The tetraphenylborate complex **7b** displays short bond lengths around the Mn^3+^ center at 100 K (Table 3), indicative of an *S* = 1 species, which is in line with the observed magnetic properties at the same temperature (Figure 4).

### Distortion Parameters

The values for Σ and Θ for mononuclear Mn^3+^ compounds are usually in the range of Σ = 28°–45° for *S* = 1 (Σ = 48°–70° for *S* = 2) and Θ = 79°–125° for *S* = 1 (Θ = 135°–230° for *S* = 2).^19,26^ Both Σ and Θ have been calculated for both series of compounds using OctaDist.^26^ The structural distortion parameters for the [Mn(3-NO_2_-5-OMe-sal_2_-323)]X complexes **3a**, **4a**, **6a** and **7a** and the [Mn(3-OMe-5-NO_2_-sal_2_-323)]X complexes **1b**–**7b** are given in Table 4. These distortion parameters are in line with the observed magnetic properties.

**Table 4:** Distortion Parameters for [Mn(3-NO_2_-5-OMe-sal_2_-323)]X Complexes **3a**, **4a**, **6a** and **7a** and the [Mn(3-OMe-5-NO_2_-sal_2_-323)]X Complexes **1b**–**7b**

Mn–X	Σ	Θ	spin state
Temp (K)	100 (>100)	100 (>100)	
Cl^–^ (**3a**)	28.75	87.46	*S* = 1
NO_3_^–^ (**4a**)	22.81^a^; 26.67^b^	68.06^a^; 75.26^b^	*S* = 1
PF_6_^–^ (**6a**)	32.75	85.33	*S* = 1
BPh_4_^–^ (**7a**)	25.56^c^; 25.56^d^	75.50^c^; 75.48^d^	*S* = 1
ClO_4_^–^ (**1b**)	45.51	142.39	*S* = 1
BF_4_^–^ (**2b**)	46.13	133.34	*S* = 1
Cl^–^ (**3b**)	37.85	112.35	*S* = 1
Br^–^ (**4b**)	41.83 (42.28)^e^	125.26 (125.46)^e^	*S* = 1 (= 1)^e^
OTf^–^ (**5b**)	81.70	290.86	*S* = 2
	71.19	266.04	*S* = 2
NTf_2_^–^ (**6b**)	36.46 (38.13)^f^	106.10 (110.90)^f^	*S* = 1 (= 1)^f^
	48.76 (74.98)^f^	140.86 (237.26)^f^	*S* = mixed (= 2)^f^
BPh_4_^–^ (**7b**)	47.61	143.42	*S* = mixed

a Polymorph a = mor780 (needle; *P*6_5_22).

b Polymorph b = mor781 (block; *C*2/*c*).

c Polymorph c = mor706: **7a**·MeOH·0.5MeCN.

d Polymorph d = mor771: **7a**·2EtOH.

e **4b** measured at 293 K.

f **6b** measured at 180 K.

Very small Σ and Θ distortion angles are found for the (**a**) series of compounds, where the nitro substituents are in the 3-position. The Σ values range between 22.8° and 32.8° and the Θ values range between 68.1° and 87.5°, which suggests that this family of compounds are all in the spin triplet state which is in line with the observed magnetic properties.

In the (**b**) series of [Mn(3-OMe-5-NO_2_-sal_2_-323)]X compounds, **1b**–**7b**, the Σ and Θ distortion angles vary depending on the spin state of the Mn^3+^ center. While compounds **1b**·0.5EtOH, **2b**·0.5MeCN, **3b** and **4b** are all found to be in the spin triplet form at 100 K (according to bond lengths and magnetic properties), the Σ values are found to be at the higher end of the *S* = 1 range and much bigger than the respective values in the (**a**) series of *S* = 1 compounds.

Within **7b**, the distortion of the complex leads to Σ and Θ values that are too large to be *S* = 1, even though the bond lengths (Table 3) and magnetic properties (Figure 4) suggest that **7b** is in the spin triplet form.

Complex **5b·**0.5H_2_O, the only complex of series (**b**) that remains HS over the entire measured temperature range, exhibits very large Σ and Θ distortions. While the Σ value of 71.2° of Mn2 would be just within the acceptable range for *S* = 1, Mn1 exhibits a Σ value of 81.7°. A similar trend is seen within the Θ distortions of **5b·**0.5H_2_O, with values of Θ = 266° and 291°.

This raises the question of whether complexes with very small Σ and Θ distortions, similar to those found in series (**a**), remain in the *S* = 1 state, while on the other hand very highly distorted complexes (like **5b·**0.5H_2_O) become trapped in the *S* = 2 state without any possibility of spin transition. We have therefore recalculated all the Σ and Θ distortion parameters using the respective cif files of previously published complexes of the [Mn(R-sal_2_-323)]X type and compare them here with those for complexes **3a**, **4a**, **6a** and **7a** and those for **1b**–**7b**. The Σ and Θ values together with the observed spin state are summarized in Table 5.

**Table 5:** Distortion Parameters, Σ and Θ, for Published [Mn(R-sal_2_-323)]X-Type Complexes Calculated with OctaDist (ref (26))^a^

Structure	Molecular Formula	*T* (K)	Spin State	Σ (deg)	Θ (deg)	ref
[Mn(sal_2_-323)]X						
1	[Mn(sal_2_-323)]Cl	293	mixed	45.9	136.1	(27)
2		100	1	35.5	103.4	(28)
3		295	mixed	46.5	137.8	(28)
4	[Mn(sal_2_-323)]SbF_6_	100	2	69.7	231.6	(28)
5		295	2	71.9	240.4	(28)
6	[Mn(sal_2_-323)]AsF_6_	110	1	31.5	93.1	(28)
7		295	2	59.1	205.3	(28)
8	[Mn(sal_2_-323)]NO_3_·EtOH	100	1	44.2	154.2	(28)
9		295	mixed	54.1	190.3	(28)
10	[Mn(sal_2_-323)]PF_6_	100	1	28.9	87.9	(29)
11		142	2	54.4	198.8	(29)
12	[Mn(sal_2_-323)]BPh_4_	240	2	68.2	231.2	(30)
13	[Mn(sal_2_-323)]ReO_4_	10	1	28.3	87.9	(31)
			1	24.1	79.1	(31)
14		80	1	35.9	138.2	(31)
			1	27.4	92.3	(31)
			2	43.6	165.0	(31)
			2	55.4	220.4	(31)
15		100	2	42.8	164.9	(31)
16		250	2	51.6	198.5	(31)
[Mn(3-OMe-sal_2_-323)]X						
17	[Mn(3-OMe-sal_2_-323)][Ni(dmit)_2_]	100	1	44.5	136.9	(32)
			1	42.0	127.0	
18		293	2	68.1	226.1	(32)
			2	64.7	216.9	
19	[Mn(3-OMe-sal_2_-323)][Pt(mnt)_2_]·2CH_3_CN	100	2	67.5	226.1	(32)
20		273	2	69.3	233.6	(32)
21	[Mn(3-OMe-sal_2_-323)]BPh_4_	100	2	78.2	287.3	(20)
22	[Mn(3-OMe-sal_2_-323)]PF_6_·H_2_O	100	1	31.9	99.8	(20)
23		293	mixed	44.9	152.7	(20)
24	[Mn(3-OMe-sal_2_-323)]ClO_4_·H_2_O	100	1	32.8	100.8	(20)
25		293	2	48.0	164.5	(20)
26	[Mn(3-OMe-sal_2_-323)]BF_4_·H_2_O	100	1	32.8	103.3	(20)
27		293	2	49.1	169.9	(20)
28	[Mn(3-OMe-sal_2_-323)]NO_3_	100	1	44.9	129.9	(18)
29		300	2	70.7	222.4	(18)
[Mn(3-OEt-sal_2_-323)]X						
30	[Mn(3-OEt-sal_2_-323)]BPh_4_	100	2	70.2	229.7	(20)
31	[Mn(3-OEt-sal_2_-323)]PF_6_	100	2	75.9	247.7	(20)
32	[Mn(3-OEt-sal_2_-323)]ClO_4_	100	1	33.9	110.4	(20)
33		293	mixed	51.3	172.3	(20)
34	[Mn(3-OEt-sal_2_-323)]BF_4_·0.4H_2_O	100	1	34.3	114.0	(20)
35		293	mixed	51.2	174.5	(20)
36	[Mn(3-OEt-sal_2_-323)]NO_3_·EtOH	100	2	66.9	252.5	(20)
37	[Mn(3-OEt-sal_2_-323)]NO_3_·0.6EtOH	293	2	68.0	244.3	(20)
[Mn(3-Me-sal_2_-323)]X						
38	[Mn(3-Me-sal_2_-323)]ClO_4_·0.31EtOH·0.15MeCN	100	1	34.7	98.9	(33)
			2	60.5	216.5	
39	[Mn(3-Me-sal_2_-323)]ClO_4_·0.25EtOH·0.13MeCN	293	2	52.6	165.4	(33)
			2	60.9	219.5	
40	[Mn(3-Me-sal_2_-323)]BF_4_·0.25EtOH·0.19MeCN	100	1	32.9	93.9	(33)
			2	61.4	222.3	
41	[Mn(3-Me-sal_2_-323)]BF_4_·0.25EtOH·0.13MeCN	293	2	50.2	158.4	(33)
			2	61.7	223.2	
42	[Mn(3-Me-sal_2_-323)]PF_6_	100	1	28.1	82.3	(33)
43		293	2	51.8	170.5	(33)
44	[Mn(3-Me-sal_2_-323)]NO_3_	100	1	42.8	125.2	(33)
45		293	2	65.3	207.4	(33)
46	[Mn(3-Me-sal_2_-323)]OTf·MeOH	100	1	33.3	93.7	(33)
47		293	2	62.6	205.4	(33)
48	[Mn(3-Me-sal_2_-323)]BPh_4_	100	1	28.1	79.4	(33)
			1	33.3	90.7	
49		293	mixed	44.8	133.5	(33)
			1	42.0	118.5	
[Mn(3-NO_2_-sal_2_-323)]X						
50	[Mn(3-NO_2_-sal_2_-323)]NO_3_	100	1	31.3	89.9	(22)
51		293	1	33.5	97.8	(22)
52	[Mn(3-NO_2_-sal_2_-323)]ClO_4_	100	1	31.6	90.6	(22)
			1	31.6	97.0	
53	[Mn(3-NO_2_-sal_2_-323)]PF_6_	100	1	35.1	102.1	(22)
54		174	mixed	44.4	131.2	(22)
55	[Mn(3-NO_2_-sal_2_-323)]OTf	100	1	28.2	83.9	(22)
			1	37.3	104.2	
56		293	mixed	45.7	164.4	(22)
57	[Mn(3-NO_2_-sal_2_-323)]BPh_4_·2MeCN	100	1	32.7	75.5	(22)
			2	99.4	259.8	
58		200	2	57.7	191.3	(22)
59	[Mn(3-NO_2_-sal_2_-323)]BPh_4_·3MeCN	100	2	50.2	161.8	(22)
60		200	2	60.0	201.5	(22)
[Mn(Nap-sal_2_-323)]X						
61	[Mn(Nap-sal_2_-323)]NTf_2_	100	1	35.3	97.2	(21)
62	[Mn(Nap-sal_2_-323)]ClO_4_	100	1	31.0	97.1	(21)
63		273	1	38.9	129.2	(34)
64	[Mn(Nap-sal_2_-323)]ClO_4_·0.5EtOH	100	2	71.4	238.8	(21)
65	[Mn(Nap-sal_2_-323)]BF_4_	100	1	31.3	88.4	(21)
66	[Mn(Nap-sal_2_-323)]BF_4_·0.5EtOH	100	2	71.3	239.2	(21)
67	[Mn(Nap-sal_2_-323)]NO_3_	100	1	34.3	96.5	(21)
68	[Mn(Nap-sal_2_-323)]SbF_6_	100	1	45.4	127.9	(34)
69		295	2	72.0	218.2	(34)
70	[Mn(Nap-sal_2_-323)]AsF_6_	100	1	50.3	141.9	(34)
			1	33.6	125.9	
			1	45.9	131.2	
71		295	2	72.5	220.4	(34)
72	[Mn(Nap-sal_2_-323)]PF_6_·0.5EtOH	100	2	67.4	225.3	(34)
73		295	2	67.9	227.4	(34)
[Mn(4-OMe-sal_2_-323)]X						
74	[Mn(4-OMe-sal_2_-323)]OTf·0.4H_2_O	100	1	30.4	87.1	(23)
75		293	mixed	45.3	140.3	(23)
76	[Mn(4-OMe-sal_2_-323)]PF_6_·0.8H_2_O	100	1	30.8	85.9	(23)
77		293	mixed	46.3	145.7	(23)
78	[Mn(4-OMe-sal_2_-323)]PF_6_·0.1sal·0.4H_2_O	100	2	72.1	274.5	(23)
79	[Mn(4-OMe-sal_2_-323)]BPh_4_	100	2	71.1	256.6	(23)
80	[Mn(4-OMe-sal_2_-323)]ClO_4_	100	2	61.7	233.8	(35)
81	[Mn(4-OMe-sal_2_-323)]BF_4_	100	2	59.6	225.9	(35)
82	[Mn(4-OMe-sal_2_-323)]NO_3_	100	mixed	53.2	202.4	(35)
		293	2	58.4	221.5	
83	[Mn(4-OMe-sal_2_-323)]Br	100	1	39.1	141.4	(35)
		190	mixed	49.7	179.8	
84	[Mn(4-OMe-sal_2_-323)]I	100	mixed	57.4	214.9	(35)
85	[Mn(4-OMe-sal_2_-323)]Cl·0.3H_2_O·3.9MeOH	100	2	56.7	230.2	(35)
[Mn(5-F-sal_2_-323)]X						
86	[Mn(5-F-sal_2_-323)]OTf	100	2	72.5	258.6	(36)
			2	63.8	217.3	
87	[Mn(5-F-sal_2_-323)]ClO_4_	100	1	29.3	91.8	(36)
88		293	2	48.9	170.9	(36)
89	[Mn(5-F-sal_2_-323)]NO_3_	293	2	61.2	189.8	(36)
90		120	1	46.1	137.7	(37)
91		298	2	61.9	191.2	(37)
92	[Mn(5-F-sal_2_-323)]PF_6_	100	2	79.8	306.8	(38)
93		298	2	81.4	311.0	(38)
94	[Mn(5-F-sal_2_-323)]AsF_6_	100	2	78.1	297.3	(38)
95		298	2	79.7	301.9	(38)
96	[Mn(5-F-sal_2_-323)]SbF_6_	100	2	75.0	280.5	(38)
97		298	2	76.8	284.9	(38)
98	[Mn(5-F-sal_2_-323)]BF_4_	100	mixed	41.3	122.2	(38)
99		298	2	58.4	184.9	(38)
100	[Mn(5-F-sal_2_-323)]ClO_4_	100	mixed	53.9	169.6	(38)
101		298	2	58.5	186.9	(38)
102	[Mn(5-F-sal_2_-323)]Cl	100	1	36.7	111.2	(38)
103		298	mixed	51.0	155.6	(38)
104	[Mn(5-F-sal_2_-323)]Cl_0.3_Br_0.7_	100	1	37.9	113.4	(38)
105		298	mixed	56.8	172.6	(38)
106	[Mn(5-F-sal_2_-323)]I	100	1	40.5	117.7	(38)
107		298	mixed	66.2	201.9	(38)
108	[Mn(5-F-sal_2_-323)]BPh_4_	100	1	39.6	120.6	(39)
109		250	2	60.2	197.4	(39)
[Mn(3,5-diF-sal_2_-323)]X						
110	[Mn(3,5-diF-sal_2_-323)]BPh_4_	100	1	34.0	106.8	(40)
111		160	mixed	48.6	160.1	(40)
112		240	2	58.5	198.8	(40)
[Mn(5-Cl-sal_2_-323)]X						
113	[Mn(5-Cl-sal_2_-323)]NO_3_	100	2	64.7	238.1	(41)
			mixed	59.5	197.5	(41)
114		293	2	65.0	227.8	(41)
			2	64.3	209.3	
115	[Mn(5-Cl-sal_2_-323)]ClO_4_	100	1	33.4	96.1	(41)
			2	66.0	229.1	
116		260	mixed	52.9	165.8	(41)
			2	66.1	231.7	
117	[Mn(5-Cl-sal_2_-323)]TNCQ_1.5_·2MeCN	100	2	65.6	211.8	(42)
118		220	2	66.8	220.1	(42)
119	[Mn(5-Cl-sal_2_-323)]BPh_4_	100	1	38.4	115.1	(43)
120		120	mixed	46.6	141.6	(43)
121		240	2	62.4	200.4	(43)
122	[Mn(5-Cl-sal_2_-323)]I	100	2	64.1	203.7	(37)
123		298	2	66.5	213.0	(37)
[Mn(3,5-diCl-sal_2_-323)]X						
124	[Mn(3,5-diCl-sal_2_-323)]NO_3_·EtOH	100	1	36.1	111.9	(41)
125		293	mixed	48.9	152.1	(41)
126	[Mn(3,5-diCl-sal_2_-323)]ClO_4_·MeOH	100	1	27.2	80.1	(41)
127	[Mn(3,5-diCl-sal_2_-323)]NTf_2_	120	1	35.3	101.7	(44)
128		185	1	37.7	109.1	(44)
			2	58.1	190.8	
129		210	mixed	48.8	149.5	(44)
130		260	2	55.9	177.9	(44)
131	[Mn(3,5-diCl-sal_2_-323)]BPh_4_	10	1	36.8	120.8	(45)
			1	39.7	120.8	
132		82	1	37.8	126.4	(45)
			1	39.3	122.1	
133		115	1	43.9	143.2	(45)
			mixed	53.4	180.5	
			2	67.0	253.8	
			2	74.6	271.9	
134		150	2	69.7	259.9	(45)
			2	63.3	237.3	
135		250	2	64.0	240.5	(45)
136		100	1	36.7	127.4	(39)
			mixed	49.3	162.9	
			2	68.0	255.9	
			2	73.7	274.0	
137		160	2	63.7	237.3	(39)
			2	69.3	259.6	
138		240	2	64.0	239.5	(39)
[Mn(5-Br-sal_2_-323)]X						
139	[Mn(5-Br-sal_2_-323)]ClO_4_	100	1	37.0	108.0	(46)
			2	66.3	227.5	
140		293	mixed	58.8	186.3	(46)
			2	65.5	225.0	
141	[Mn(5-Br-sal_2_-323)]NO_3_	293	2	64.4	232.9	(41)
142	[Mn(5-Br-sal_2_-323)]PF_6_	293	2	74.7	251.2	(41)
143	[Mn(5-Br-sal_2_-323)]TNCQ_1.5_·2MeCN	100	2	67.0	221.9	(42)
144	Mn(5-Br-sal_2_-323)][Ni(mnt)_2_]	123	1	19.5	71.3	(47)
145		296	1	24.2	92.5	(47)
146		350	1	29.5	113.3	(47)
147		473	mixed	37.6	146.7	(47)
148	Mn(5-Br-sal_2_-323)][Pt(mnt)_2_]	400	mixed	37.1	137.1	(47)
149		473	mixed	40.3	153.2	(47)
150	Mn(5-Br-sal_2_-323)][Ni(dmit)_2_]·MeCN	296	2	83.6	315.5	(47)
151	[Mn(5-Br-sal_2_-323)]BPh_4_	100	1	35.1	101.9	(30)
152		120	mixed	40.5	118.9	(30)
153		240	2	61.1	195.6	(30)
154	[Mn(5-Br-sal_2_-323)]I	100	1	31.1	92.7	(37)
155		298	mixed	37.2	110.7	(37)
[Mn(3,5-diBr-sal_2_-323)]X						
156	[Mn(3,5-diBr-sal_2_-323)]BPh_4_	25	1	34.9	117.7	(48)
			2	76.4	287.9	
			2	69.2	263.6	
			1	43.1	143.1	
157		110	2	67.7	259.9	(48)
			2	73.4	278.9	
158		293	2	66.5	251.5	(48)
159	[Mn(3,5-diBr-sal_2_-323)]BF_4_·EtOH	100	1	36.7	113.4	(19)
160		293	2	62.0	215.9	(19)
161	[Mn(3,5-diBr-sal_2_-323)]OTf·EtOH	100	1	29.7	192.6	(19)
162		293	2	59.6	219.9	(19)
163	[Mn(3,5-diBr-sal_2_-323)]NO_3_·EtOH	100	1	27.4	79.5	(19)
164	[Mn(3,5-diBr-sal_2_-323)]PF_6_·MeOH	100	1	25.4	75.5	(19)
165	[Mn(3,5-diBr-sal_2_-323)]ClO_4_·EtOH	100	mixed	47.2	152.2	(19)
166		293	2	64.7	228.3	(19)
167	[Mn(3,5-diBr-sal_2_-323)]ClO_4_·0.5MeCN	100	1	24.0	74.8	(19)
168		293	1	36.8	119.2	(19)
[Mn(3,5-Br,F-sal_2_-323)]X						
169	[Mn(3,5-Br,F-sal_2_-323)]BPh_4_ (monoclinic)	100	1	35.4	106.2	(40)
170		160	mixed	55.5	175.4	(40)
171		240	2	64.6	210.1	(40)
172	[Mn(3,5-Br,F-sal_2_-323)]BPh_4_ (triclinic)	100	2	69.8	260.8	(40)
			2	66.8	230.7	
173		160	2	69.5	258.9	(40)
			2	68.1	235.0	
174		240	2	70.4	259.4	(40)
			2	68.3	237.4	
[Mn(3,5-Br,Cl-sal_2_-323)]X						
175	[Mn(3,5-Br,Cl-sal_2_-323)]BPh_4_	100	2	68.5	262.9	(39)
			2	73.3	276.7	
176		240	2	65.3	249.9	
[Mn(5-I-sal_2_-323)]X						
177	[Mn(5-I-sal_2_-323)]I	100	1	32.2	92.4	(37)
178		298	mixed	39.6	121.1	
179	[Mn(5-I-sal_2_-323)]NO_3_	100	mixed	39.6	117.1	(37)
			1	33.6	99.6	
180		298	mixed	40.0	118.6	
			mixed	40.2	116.9	
[Mn(5-NO_2_-sal_2_-323)]X						
181	[Mn(5-NO_2_-sal_2_-323)]NO_3_·MeCN·0.5H_2_O	100	1	30.9	103.6	(22)
			2	78.4	270.0	
182		293	mixed	44.5	159.5	(22)
			2	80.7	284.6	
183	[Mn(5-NO_2_-sal_2_-323)]ClO_4_·MeCN·0.5H_2_O	100	1	29.6	87.2	(22)
			2	95.2	304.7	
184		260	1	36.0	87.8	(22)
			2	113.1	312.5	
185	[Mn(5-NO_2_-sal_2_-323)]ClO_4_·0.5MeCN·0.5H_2_O	100	2	87.3	329.0	(22)
186	[Mn(5-NO_2_-sal_2_-323)]PF_6_	100	1	31.3	90.3	(22)
			1	35.9	110.1	
187	[Mn(5-NO_2_-sal_2_-323)]OTf	100	2	65.8	251.3	(22)
188	[Mn(5-NO_2_-sal_2_-323)]BPh_4_	100	2	77.7	280.4	(22)
[Mn(5-azo-sal_2_-323)]X						
189	[Mn(5-azo-sal_2_-323)]Cl	100	1	36.1	106.2	(49)
190		240	1	37.4	109.8	
191	[Mn(5-azo-sal_2_-323)]BF_4_·2MeCN	100	2	66.8	239.0	(49)
192	[Mn(5-azo-sal_2_-323)]ClO_4_·1MeCN	100	2	69.5	257.3	(49)
193	[Mn(5-azo-sal_2_-323)]PF_6_	100	2	68.8	262.3	(49)
[Mn(5-OMe-sal_2_-323)]X						
194	[Mn(5-OMe-sal_2_-323)]ClO_4_	100	1	31.9	92.9	(50)
			2	64.7	204.0	
195		293	mixed	44.9	136.4	(50)
			2	64.1	210.2	
196	[Mn(5-OMe-sal_2_-323)]BF_4_	100	2	71.4	236.0	(50)
197		293	2	74.3	245.3	(50)
198	[Mn(5-OMe-sal_2_-323)]NO_3_	100	2	69.6	230.4	(50)
199		293	2	71.3	236.9	(50)
200	[Mn(5-OMe-sal_2_-323)]OTf	100	2	71.6	240.0	(50)
201		293	2	72.1	239.2	(50)
202	[Mn(5-OMe-sal_2_-323)]Cl	100	1	34.0	97.8	(51)
203		290	mixed	46.9	140.1	(51)
204		400	2	59.2	185.2	(51)
[Mn(5-OCF_3_-sal_2_-323)]X						
205	[Mn(5-OCF_3_-sal_2_-323)](*R*,*R*)spiroborate (Λ)	100	1	41.4	123.8	(52)
206		293	mixed	48.9	151.0	(52)
207	[Mn(5-OCF_3_-sal_2_-323)](*S*,*S*)spiroborate (Δ)	100	1	40.4	121.5	(52)
208		293	mixed	45.8	140.9	(52)
[Mn(4,6-diOMe-sal_2_-323)]X						
209	[Mn(4,6-diOMe-sal_2_-323)]ClO_4_·0.5H_2_O	100	1	33.4	99.9	(13)
210	[Mn(4,6-diOMe-sal_2_-323)]NO_3_·1.15H_2_O	100	1	32.9	101.1	(13)
211	[Mn(4,6-diOMe-sal_2_-323)]BF_4_·0.85H_2_O	100	1	33.6	101.0	(13)
212	[Mn(4,6-diOMe-sal_2_-323)]OTf	100	1	28.6	82.1	(13)
213	[Mn(4,6-diOMe-sal_2_-323)]Cl·EtOH	100	1	33.9	97.2	(13)
[Mn(3-NO_2_-5-OMe-sal_2_-323)]X series (**a**)						
214	[Mn(3-NO_2_-5-OMe-sal_2_-323)]Cl (**3a**)	100	1	28.8	87.5	
215	[Mn(3-NO_2_-5-OMe-sal_2_-323)]NO_3_ (**4a**)	100	1	22.8	68.1	
216	polymorph of (**4a**)	100	1	26.7	75.3	
217	[Mn(3-OMe-5-NO_2_-sal_2_-323)]PF_6_ (**6a**)	100	1	32.8	85.3	
218	[Mn(3-NO_2_-5-OMe-sal_2_-323)]BPh_4_·0.5MeCN·MeOH (**7a**)	100	1	25.6	75.5	
219	[Mn(3-NO_2_-5-OMe-sal_2_-323)]BPh_4_·2EtOH (**7a**)	100	1	25.6	75.5	
[Mn(3-OMe-5-NO_2_-sal_2_-323)]X series (**b**)						
220	[[Mn(3-OMe-5-NO_2_-sal_2_-323)]ClO_4_ (**1b**)	100	1	45.5	142.4	
221	[Mn(3-OMe-5-NO_2_-sal_2_-323)]BF_4_ (**2b**)	100	1	46.1	133.3	
222	[Mn(3-OMe-5-NO_2_-sal_2_-323)]Cl (**3b**)	100	1	37.9	112.4	
223	[Mn(3-OMe-5-NO_2_-sal_2_-323)]Br (**4b**)	100	1	41.8	125.3	
224		293	1	42.3	125.5	
225	[Mn(3-OMe-5-NO_2_-sal_2_-323)]OTf (**5b**)	100	2	81.7	290.9	
			2	71.2	266.0	
226	[Mn(3-OMe-5-NO_2_-sal_2_-323)]NTf_2_ (**6b**)	100	1	36.5	106.1	
			mixed	48.8	140.9	
227		180	1	38.1	110.9	
			2	75.0	237.3	
228	[Mn(3-OMe-5-NO_2_-sal_2_-323)]BPh_4_ (**7b**)	100	mixed	47.6	143.4	

a Spin states were assigned according to χ*T* data and bond lengths.

**Figure 6:**
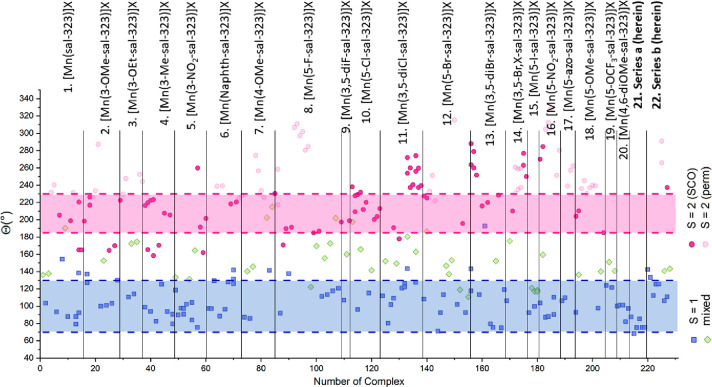
Θ values for all published (at time of writing) Mn^3+^ structures of the [Mn(R-sal_2_-323)]X-type divided by ligands, (Table 5). Structures with Mn^3+^ in the spin triplet form are displayed as blue squares; Mn^3+^ in the spin quintet form is shown as pink circles, where it is differentiated whether the compound can undergo spin transition (bold) or remains in the *S* = 2 state (pale); and Mn^3+^ complexes that cannot clearly be assigned *S* = 1 or *S* = 2 are shown as pale green diamonds.

The distortion values of Θ, derived from Table 5, are shown in Figure 6. The values found for all mononuclear Mn^3+^ compounds range between 68.1° and 329.0° highlighting the flexibility of the ligand. A close evaluation of all 228 previously reported structures (at the time of writing) and the 15 structures herein show that a structural boundary between the pure spin triplet and the pure spin quintet form within Mn^3+^ complexes can be discerned. Instead of Θ = 79°–125° for *S* = 1 and Θ = 135°–230° for *S* = 2,^20,33^ as mentioned before, these borders should be revised to Θ = 68°–130° for the spin triplet form and Θ = 185°–230° for the spin quintet form. In the majority of cases where Θ > 230°, the complexes remain in the spin quintet form and do not undergo thermal spin transition in the solid state. This can often be observed also for complexes that exhibit two sites, with one site remaining in the *S* = 2 spin state over the entire measured temperature range. The strong Jahn–Teller distortion, leading to Θ > 230°, will most likely preferably keep the complex in the spin quintet form and will prevent the possibility of switching to the spin paired triplet form.

There are a few exceptions to this: [Mn(3-NO_2_-sal-323)]BPh_4_·2MeCN,^22^ [Mn(3,5-diCl-sal-323)]BPh_4_,^39,45^ [Mn(3,5-diBr-sal-323)]BPh_4_,^48^ [Mn(3,5-Br,X-sal-323)]BPh_4_,^39,40^ and [Mn(3-OMe-5-NO_2_-sal_2_-323)]NTf_2_ (**6b**). All of these exhibit more than one unique Mn^3+^ site in the crystal structure as well as sterically demanding substituents on the ligand and also contain a large counterion. In case of [Mn(3-NO_2_-sal-323)]BPh_4_·2MeCN,^22^ the large Θ value of 259.8° is only observed within one Mn^3+^ site at 100 K, before the crystal undergoes a phase transition and at 200 K a normal Θ value of 191.3° for one single site is observed.

**Figure 7:**
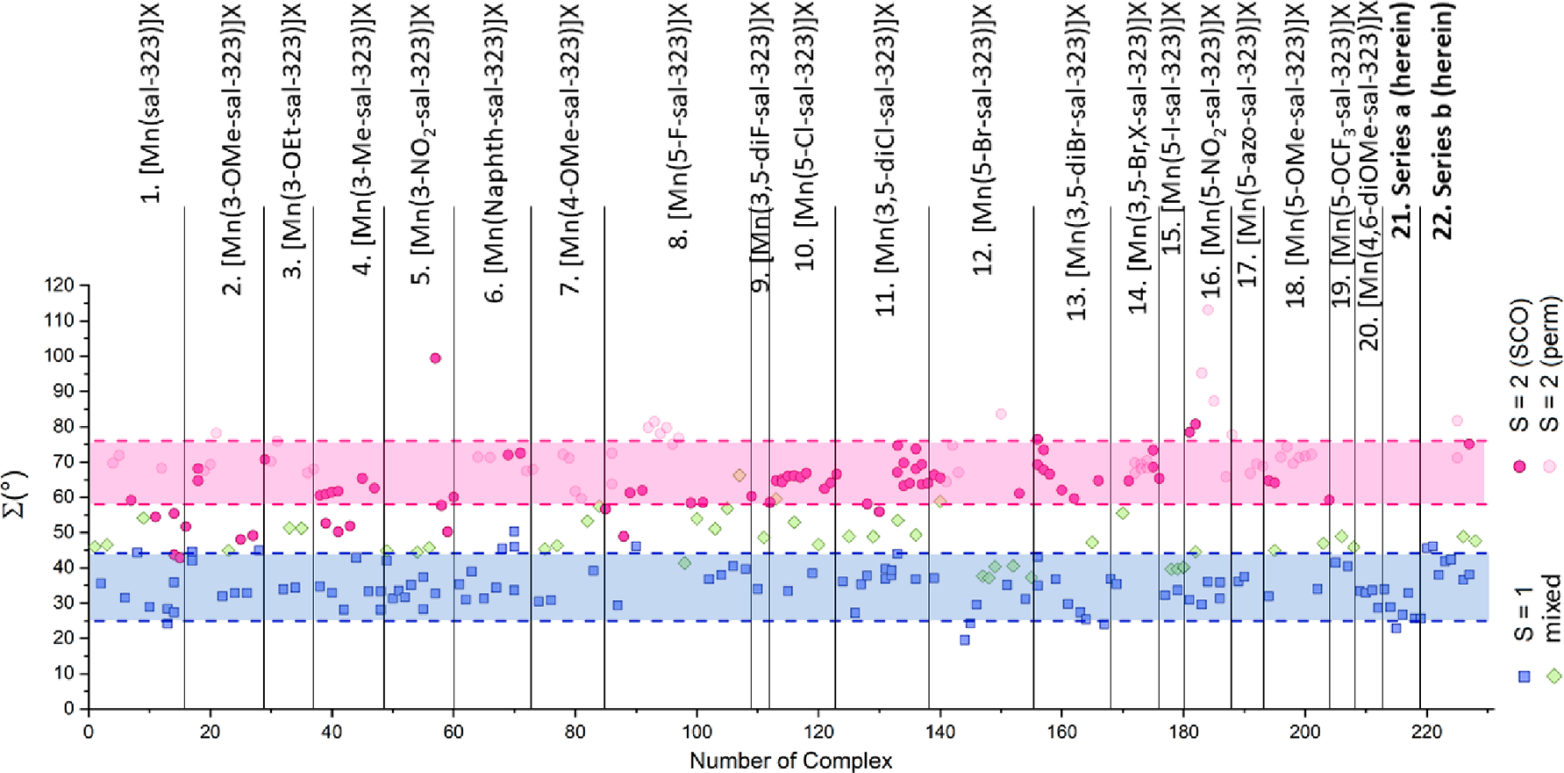
Σ values for all published (at time of writing) Mn^3+^ structures of the [Mn(R-sal_2_-323)]X-type divided by ligands (Table 5). Structures with Mn^3+^ in the spin triplet form are displayed as blue squares; Mn^3+^ in the spin quintet form is shown as pink circles, where it is differentiated whether the compound can undergo spin transition (bold) or remains in the *S* = 2 state (pale); and Mn^3+^ complexes that cannot clearly be assigned *S* = 1 or *S* = 2 are shown as pale green diamonds.

The distortion values of Σ, derived from Table 5, are shown in Figure 7. The Σ values found for all 228 mononuclear Mn^3+^ structures show a narrower range than in case of the Θ values. The Σ values were found to be between 19.5° and 113.1°, with a clear border between the pure spin triplet and the pure spin quintet form. Instead of Σ = 28°–45° for *S* = 1 and Σ = 48°–70° for *S* = 2, as mentioned before,^20,33^ these borders should be revised to Σ = 20°–45° for the spin triplet form and Θ = 58°–76° for the spin quintet form. The area between the spin triplet and the spin quintet complexes is smaller than in the case of the Θ values (Figure 6), making the border harder to define, leading to a bigger overlap with complexes that are neither fully in the *S* = 1 state nor in the *S* = 2 state.

Interestingly, some complexes with really small Σ values (Σ < 25°) have been observed, such as [Mn(5-Br-sal-323)][Ni(mnt)_2_],^47^ and [Mn(3,5-diBr-sal-323)]ClO_4_·0.5MeCN,^19^ as well as **4a** and **7a**. The small angular distortions from the idealized octahedral environment around the Mn^3+^ centers, with Σ < 26° seem to trap the complex in the spin triplet state, at least in the solid state. A spin transition to the Jahn–Teller distorted spin quintet form would require energy and space for the distortion around the Mn^3+^ center which is only likely to happen at much higher temperatures.

### Twist Angles of the NO_2_ Substituents

**Figure 8:**
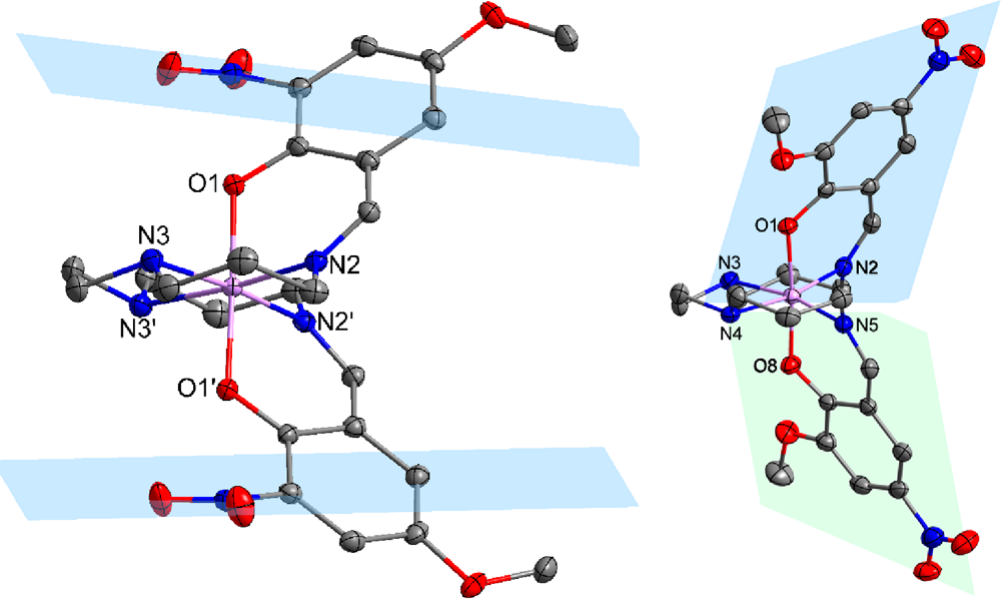
Twist of the NO_2_ groups out-of-plane in respect to the phenol ring of the Schiff base ligand of the cationic Mn^3+^ species of **4a** (left) and the in-plane arrangement of **1b** (right).

We have recently investigated spin state choices in Mn^3+^ with two ligands closely related to L1 and L2, those with nitro groups appended either in the *ortho* or *para* positions of the ligand phenolate donors.^22^ Although we had initially considered a correlation between the electronic character of the ligand and choice of spin state, computational analysis suggested little difference between the ligand field strengths of the two ligands. In the earlier work we have therefore ascribed the tendency of the ligand with nitro groups in the *ortho*-position to stabilize the spin triplet form of Mn^3+^ as mainly due to packing effects. As part of our analysis in the earlier paper and also in the current work, we have measured the out-of-plane angle of the nitro groups relative to the phenolate donors. The ligands used in the current study are similar to those used in the earlier report in that there are nitro groups in the 3 or 5 positions of the phenolate ring,^22^ but here they also bear an additional methoxy group, which should not interfere with the flexibility and the degree of torsional distortion of the nitro groups. The degrees of conjugation of the nitro substituents relative to the phenol rings in the two complex series are compared in Table 6 and could be considered to show some correlation to the choice of spin state. This analysis highlights the same but much clearer trend as reported in ref (22): (i) the nitro group of the Schiff base ligand is flipped out-of-plane for most of the complexes with the *ortho* nitro-substitution, series (**a**); and (ii) those with the *para* nitro-substituted ligands, series (**b**), are mostly in-plane with the benzene ring of the respective Schiff base (Figure 8).

**Table 6:** Angle between the Plane of the NO_2_ Substituent and the Benzene Ring of the Schiff Base Ligand for [Mn(3-NO_2_-5-OMe-sal_2_-323)]X complexes **3a**, **4a**, **6a** and **7a** and the [Mn(3-OMe-5-NO_2_-sal_2_-323)]X Complexes **1b**–**7b**

Mn–X	Sal1–NO_2_	Sal2–NO_2_
Temp (K)	100 (>100)	100 (>100)
Cl^–^ (**3a**)	33.1°	33.1°
NO_3_^–^ (**4a**)	46.2^a^; 8.6^b^	46.2^a^; 8.6^b^
PF_6_^–^ (**6a**)	12.9°	9.9°
BPh_4_^–^ (**7a**)	47.2^c^; 51.2^d^,^g^	20.5^c^; 21.1^d^
ClO_4_^–^ (**1b**)	5.3°	3.8°
BF_4_^–^ (**2b**)	3.6°	5.7°
Cl^–^ (**3b**)	11.9°	11.9°
Br^–^ (**4b**)	13.2° (12.7°)^e^	13.2° (12.7°)^e^
OTf^–^ (**5b**)	7.3°	12.0°
	2.9°	7.4°
NTf_2_^–^ (**6b**)	13.1° (13.1°)^f^	17.1° (15.6°)^f^
	16.3° (16.2°)^f^	5.9° (2.2°)^f^
BPh_4_^–^ (**7b**)	8.3°	5.7°

a Polymorph a = mor780 (needle; *P*6_5_22).

b Polymorph b = mor781 (block; *C*2/*c*).

c Polymorph c = mor706:7a·MeOH·0.5MeCN.

d Polymorph d = mor771: **7a**·2EtOH.

e **4b** measured at 293 K

f **6b** measured at 180 K.

g **7a** exhibits a disorder of both oxygen atoms of the NO_2_ group, O2 and O3. The two positions are crystallographically weighted 0.92:0.08 leading to angles of 48.6°:81.1°, and the weighted average is used.

Within series (**a**), the angles between the plane of the NO_2_ group in the 3-position and the benzene ring of the Schiff base ligand of complexes **3a**, **4a** and **7a** are in the range between 20.5° and 51.2° (see Table 6), highlighting the out-of-plane character of the nitro substituents. The PF_6_^–^ containing compound, **6a**, exhibits the smallest twist angles of 9.9° and 12.9°, respectively. Also, one of the polymorphs of complex **4a** was found to have its nitro substituents almost in plane, with twisting angles of 8.6° for both nitro groups.

Within series (**b**) on the other hand, the angles between the plane of the NO_2_ group in the 5-position and the benzene ring of the Schiff base ligand are found to be smaller than 10° in the majority of cases. Only the halide containing compounds, **3b** and **4b**, exhibit twisting angles between 11.9° and 13.2°, as well as complex **6b**, containing the bistriflimide anion, which shows the largest twisting angles at 100 and 180 K, respectively (see Table 6). A clear trend emerges for preference for the spin triplet form of the coordinated manganese when the ligand nitro group is *ortho* to the phenolate oxygen in the L1 complexes. This may be due to an electronic effect of the nitro donor but changes in the packing associated with the position of the ligand substituents may also play a role here.

## Conclusions

In this report we have demonstrated a correlation between ligand substituent and spin state choice for two cationic [Mn(R-sal_2_323)]^+^ complexes in different lattices. The Mn^3+^ complex with L1, which has *ortho*-appended nitro groups and *para*-appended methoxy groups, was stabilized in the spin triplet form in six different lattices, and in a seventh (BPh_4_^–^) when solvated. Loss of solvent facilitated an irreversible change to the HS *S* = 2 form, suggesting that packing effects, rather than electronic influences, dominate the choice of spin state. On the other hand, the Mn^3+^ complex of the isomeric ligand L2 with *ortho*-appended methoxy groups and *para*-appended nitro groups could be stabilized in spin triplet and spin quintet forms over the same temperature range when embedded in a variety of lattices, also exhibiting thermal SCO in some cases. Given the similar electronic character of the two ligands L1 (3-NO_2_-5-OMe-sal_2_323) and L2 (3-OMe-5-NO_2_-sal_2_323), it is likely that differences in the packing of the coordinated cations constitute the major drivers of choice of spin state in the solid-state forms of the complex families. This conclusion is supported by comparison with earlier published examples of Mn^3+^ complexes with related nitro-substituted ligands where computational analysis showed little difference between the strengths of the local crystal fields and differences in spin state choices were attributed to packing.^22^ However, it is not possible to rule out an electronic contribution from structural data alone. We have also compared the bond length and angular distortion data of the structures reported here with earlier reported examples of [Mn(R-sal_2_323)]^+^ complexes, in an effort to relate the degree of distortion with the ability to switch spin state over a thermal range. We suggest that a barrier to switching from HS to LS exists for those spin quintet complexes which are particularly distorted at room temperature, as they are observed to remain HS on cooling. It is less clear if there is a barrier to switching from LS to HS, as all the spin triplet forms start to show a thermal SCO on warming. It seems likely that the pressure of crystal packing is necessary to stabilize the LS forms of Mn^3+^ in the generic family of [Mn(R-sal_2_323)]^+^ complexes, and we continue to test this idea by isolating related complexes in different material forms to study their properties beyond the crystalline state.

## Experimental Section

### General Experimental

Physical measurements: All measurements were carried out on powdered samples of the respective polycrystalline compound. Elemental analyses (C, H, and N) were performed using a PerkinElmer Vario EL. A Bruker Alpha Platinum FTIR spectrometer was used to record the infrared spectra in reflectance mode.

### Materials and Synthetic Procedures

#### Starting Materials

All chemicals and solvents if not otherwise mentioned were purchased from chemical companies and were reagent grade. They were used without further purification or drying. All reactions were carried out under ambient conditions. All measurements were carried out on powdered samples of the respective polycrystalline compound.

#### Synthesis and Characterization of Series **a**, Complexes **1a**–**7a**

##### Series (**a**) (3-NO_2_-5-OMe-sal_2_-323)

To a solution of *N*,*N*′-bis(3-aminopropyl)ethylenediamine (174 mg, 1.0 mmol) in a 50:50 solution of acetonitrile:ethanol (30 mL) was added 2-hydroxy-5-methoxy-3-nitrobenzaldehyde (396 mg, 2.0 mmol) causing a color change to deep red. The respective manganese(III) salt (1.0 mmol) was added resulting in a color change to dark brown. After stirring and gentle heating for 2 h the solution was gravity filtered and left standing. Once crystals formed, they were collected, washed and analyzed.

##### **1a**

Mn(ClO_4_)_2_·6H_2_O (362 mg, 1.0 mmol) was directly used for the synthesis. Elemental analysis for C_24_H_30_N_6_O_12_ClMn (**1a**), calculated: C - 42.09, H - 4.41, N - 12.27; found: C - 41.85, H - 4.23, N - 12.09.

##### **2a**

MnCl_2_·6H_2_O (198 mg, 1.0 mmol) together with NH_4_BF_4_ (105 mg, 1.0 mmol) were used for the synthesis. Elemental analysis for **2a**·0.4EtOH·0.4MeCN (C_24_H_30_BF_4_MnN_6_O_8_·0.4C_2_H_6_O· 0.4C_2_H_3_N), calculated: C - 43.48, H - 4.79, N - 12.68; found: C - 43.63, H - 4.64, N - 12.54.

##### **3a**

MnCl_2_·4H_2_O (197 mg, 1.0 mmol) was used for the reaction. There was insufficient sample recovered to measure the C, H and N content so only the structure of compound **3a** is reported here.

##### **4a**

Mn(NO_3_)_2_·4H_2_O (251 mg, 1.0 mmol) was used for the synthesis. Elemental analysis calculated for C_24_H_30_N_7_O_11_Mn (**4a**): C - 44.52, H - 4.67, N - 15.14; found: C - 44.57, H - 4.61, N - 15.07.

##### **5a**

MnCl_2_·6H_2_O (197 mg, 1.0 mmol) and lithium trifluoromethanesulfonate (LiCF_3_SO_3_) (156 mg, 1.0 mmol) were used for the synthesis. Elemental analysis for **5a**·0.95H_2_O·0.25MeCN (C_25_H_30_F_3_MnN_6_O_11_S·0.95H_2_O·0.25C_2_H_3_N), calculated: C - 40.20, H - 4.32, N - 11.49; found: C - 39.92, H - 4.04, N - 11.75.

##### **6a**

MnCl_2_·6H_2_O (197 mg, 1.0 mmol) and KPF_6_ (184 mg, 1.0 mmol) were used. Elemental analysis for **6a**·0.55EtOH·1.25MeCN (C_24_H_30_F_6_MnN_6_O_8_P·0.55C_2_H_6_O·1.25C_2_H_3_N), calc.: C - 41.07, H - 4.63, N - 12.58; found: C - 41.45, H - 4.26, N - 12.24.

##### **7a**·MeOH·0.5MeCN

MnCl_2_·6H_2_O (197 mg, 1.0 mmol) and sodium tetraphenylborate, NaBPh_4_, (342 mg, 1.0 mmol) were used in methanol/acetonitrile. Elemental analysis for **7a**·1.85MeOH (C_48_H_50_BMnN_6_O_8_·1.85CH_4_O), calculated: C - 62.11, H - 6.00, N - 8.72; found: C - 61.90, H - 5.82, N - 8.92.

#### Synthesis and Characterization of Series **b**, Complexes **1b**–**7b**

##### Series (**b**) (3-OMe-5-NO_2_-sal_2_-323)

To a solution of *N*,*N*′-bis(3-aminopropyl)ethylenediamine (174 mg, 1.0 mmol) in a 50:50 solution of acetonitrile:ethanol (30 mL) was added solid 2-hydroxy-3-methoxy-5-nitrobenzaldehyde (396 mg, 2.0 mmol) causing a color change to deep red. The respective manganese(III) salt (2.0 mmol) was added resulting in a color change to dark brown. After stirring and gentle heating for 2 h the solution was gravity filtered and left standing until dark brown crystals formed after 2–3 days.

##### **1b**·0.5EtOH

Mn(ClO_4_)_2_·6H_2_O (362 mg, 1.0 mmol) was used for the synthesis. Elemental analysis for **1b·**0.25EtOH (C_24_H_30_ClMn N_6_O_12_·0.25C_2_H_6_O) calculated: C - 42.25, H - 4.56, N - 12.07; found: C - 42.36, h - 4.35, N - 11.85.

##### **2b**·0.5MeCN

MnCl_2_·6H_2_O (198 mg, 1.0 mmol) together with NH_4_BF_4_ (105 mg, 1.0 mmol) were used for the synthesis. Elemental analysis for **2b**·0.3MeCN (C_24_H_30_BF_4_MnN_6_O_8_·0.3 CH_3_CN), calculated: C - 43.16, H - 4.55, N - 12.89; found: C - 43.08, H - 4.53, N - 13.01.

##### **3b**

MnCl_2_·4H_2_O (197 mg, 1.0 mmol) was used for the reaction. Elemental analysis calculated for C_24_H_30_ClMnN_6_O_8_ (**3b**): C - 46.42, H - 4.87, N - 13.53; found: C - 46.42, H - 4.43, N - 13.08.

##### **4b**

MnBr_2_ (215 mg, 1.0 mmol) was used for the reaction. Elemental analysis calculated for C_24_H_30_N_6_O_8_MnBr (**4b**): C - 43.32, H - 4.54, N - 12.63; found: C - 43.24, H - 4.48, N - 12.45.

##### **5b**·0.5H_2_O

MnCl_2_·6H_2_O (197 mg, 1.0 mmol) and lithium trifluoromethanesulfonate (LiCF_3_SO_3_) (156 mg, 1.0 mmol) were used for the synthesis. Elemental analysis for **5b**·0.45H_2_O·0.35EtOH (C_25_H_30_F_3_MnN_6_O_11_S·0.45H_2_O·0.35C_2_H_6_O), calculated: C - 40.68, H - 4.38, N - 11.08; found: C - 40.38, H - 4.07, N - 10.78.

##### **6b**

MnCl_2_·6H_2_O (197 mg, 1.0 mmol) and lithium bis(trifluoro-methanesulfonyl)imide (LiN(CF_3_SO_3_)_2_) (287 mg, 1.0 mmol) were used for the synthesis. Elemental analysis for C_26_H_30_N_7_O_12_F_6_ S_2_Mn (**6b**), calculated: C - 36.08, H - 3.49, N - 11.33; found: C - 36.23, H - 3.37, N - 11.07.

##### **7b**

MnCl_2_·6H_2_O (197 mg, 1.0 mmol) and sodium tetraphenylborate, NaBPh_4_, (342 mg, 1.0 mmol) were used. Elemental analysis for C_48_H_50_BN_6_O_8_Mn (**7b**), calculated: C - 63.73, H - 5.57, N - 9.29; found: C - 63.99, H - 5.60, N - 9.14.

### Crystallography

Suitable single crystals of complexes were mounted on Oxford Diffraction Supernova E diffractometer fitted with an Atlas detector; data sets were measured using monochromatic Cu–Kα or Mo–Kα radiation and were corrected for absorption.^53^ The temperature (100 and 293 K, respectively) was controlled with an Oxford Cryosystem instrument. Structures were solved by dual-space direct methods (SHELXT)^54^ and refined with full-matrix least-squared procedures based on *F*^*2*^, using SHELXL-2016. Nonhydrogen atoms were refined with independent anisotropic displacement parameters, organic H atoms (i.e., bonded to C) were placed in idealized positions, while the coordinates of H atoms bonded to O were generally refined with their O–H distance restrained to 0.88(4) Å. Selected crystallographic data and structure refinements are summarized in Tables S-A1, S-A2, S-B1–B3, and crystallographic data for the structures reported in this paper have been deposited with the Cambridge Crystallographic Data Centre as supplementary publication numbers: CCDC 2170391–2170405 and copies of the data can be obtained free of charge from https://www.ccdc.cam.ac.uk/structures/.

### Magnetic Measurements

The magnetic susceptibility measurements were recorded on a Quantum Design SQUID magnetometer MPMS-XL on polycrystalline samples wrapped in gelatin capsules in an applied field of 1000 or 5000 Oe.

## Supporting Information Available

Additional data and details (PDF)
