# Supplementary material for: Compressed and Expanded Lattices - Barriers to Spin-State Switching in Mn3+ Complexes
Source: Cryst Growth Des. 2023 May 24;23(6):3996–4012. doi: 10.1021/acs.cgd.2c01284 (PMC10251414; doi:10.1021/acs.cgd.2c01284)
Supplement: Supplementary file 1 — cg2c01284_si_001.pdf [file cg2c01284_si_001.pdf]

Compressed and Expanded Lattices - Barriers to Spin State Switching in Mn^{3+} complexes

Michelle M. Harris,[a] Irina A. Kühne,[a,b] Conor T. Kelly,[a] Vibe B. Jakobsen,[a] Ross Jordan,[c] Luke O'Brien,[a] Helge Müller-Bunz,[a] Solveig Felton[c] and Grace G. Morgan[a,]**

Addresses:

[a] School of Chemistry, University College Dublin, Belfield, Dublin 4, Ireland.

[b] Department of Functional Materials, FZU - Institute of Physics - Czech Academy of Sciences, Na Slovance 1999/2, Prague 8, 182 21, Czech Republic.

[c] Centre for Quantum Materials and Technologies, School of Mathematics and Physics, Queen's University Belfast, BT7 1NN, United Kingdom.

Email: grace.morgan@ucd.ie

ESI

Content

Figure S1. First derivative of the χ_MT product of complex **6b**........ 2

Figure S2. Plots of $\chi_M T$ versus T for two independent samples of [Mn(3-OMe-5-NO_2-sal_2-323)]BPh_4 ,**6b**, in the temperature range 5 - 300 K in cooling mode using an applied dc field of 1000 Oe and 5000 Oe, respectively........ 3

Table A1. Crystallographic details for complexes (**3a**) – (**4a**) using 3-OMe-5-NO_2-sal_2-323 as ligand. 3

Table A2. Crystallographic details for complexes (**6a**) – (**7a**) using 3-OMe-5-NO_2-sal_2-323 as ligand. 4

Table B1. Crystallographic details for complexes (**1b**) – (**3b**) using 3-NO_2-5-OMe-sal_2-323 as ligand. 5

Table B2. Crystallographic details for complexes (**4b**) – (**5b**) using 3-NO_2-5-OMe-sal_2-323 as ligand. 6

Table B3. Crystallographic details for complexes (**6b**) – (**7b**) using 3-NO_2-5-OMe-sal_2-323 as ligand. 6

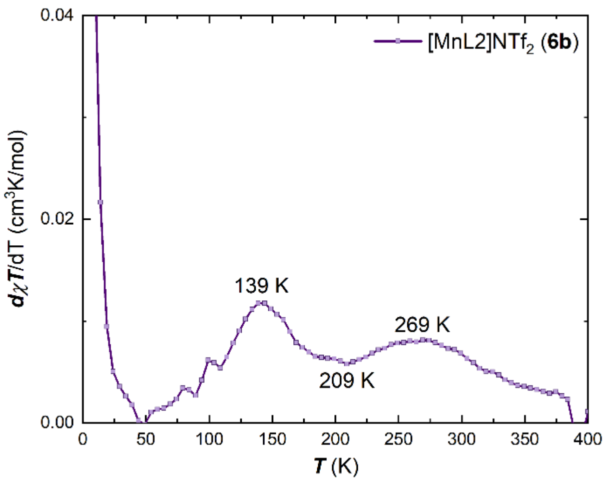

Figure S1. First derivative of the χ_MT product of complex **6b**.

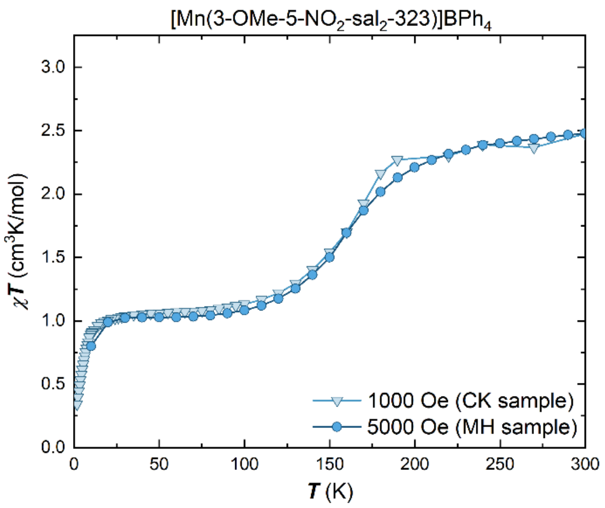

Figure S2. Plots of $\chi_M T$ versus T for two independent samples of [Mn(3-OMe-5-NO_2-sal_2-323)]BPh_4 ,**6b**, in the temperature range 5 - 300 K in cooling mode using an applied dc field of 1000 Oe and 5000 Oe, respectively.

Table A1. Crystallographic details for complexes (**3a**) – (**4a**) using 3-NO_2-5-OMe-sal_2-323 as ligand.

Compound	(**3a**)	(**4a**) needle	(**4a**) block
X^-	Cl^-	NO_3^-	NO_3^-
sample code	CCDC 2170399	CCDC 2170396	CCDC 2170395
Empirical formula	$C_{24}H_{30}N_6O_8ClMn$	$C_{24}H_{30}N_7O_{11}Mn$	$C_{24}H_{30}N_7O_{11}Mn$
Formula weight	620.93	647.49	647.49
Temperature (K)	100(2)	100(2)	100(2)
Radiation	Cu-Kα	Cu-Kα	Mo-Kα
Crystal system	monoclinic	hexagonal	monoclinic
Space group	P2/c (#13)	$P6_522$ (#179)	C2/c (#15)
Crystal size (mm)	0.231 x 0.182 x 0.076	0.262 x 0.029 x 0.018	0.261 x 0.171 x 0.159
a (Å)	7.2363(2)	9.51578(9)	12.1881(3)
b (Å)	9.3576(4)	9.51578(9)	11.9597(3)
c (Å)	19.1016(7)	52.4814(5)	17.8209(5)
α (°)	90	90	90
β (°)	91.306(3)	90	106.190(3)
γ (°)	90	120	90
V ($Å^3$)	1293.12(8)	4115.52(7)	2494.66(11)
Z	2	6	4
d_{calc} (g cm^{-3})	1.595	1.568	1.724
μ (mm^{-1})	5.643	4.568	0.611
F(000)	644	2016	1344
Limiting indices	h=±9, k=±11, l=±23	h=±11, k=±11, l=±65	h=±15, K=±15, l=±22
Reflect. coll./ uniq.	6243/2688	35185/2883	23623/2654

R(int)	0.0276	0.0403	0.0382
Complete to Θ (%)	99.9	99.7	99.8
Data/restr./param.	2688 / 0 / 183	2883 / 0 / 201	2654 / 0 / 201
GooF on F^2	1.043	1.072	1.050
Final R indices [I > 2σ(I)][a]	R_1 = 0.0360, wR_2 = 0.0970	R_1 = 0.0249, wR_2 = 0.0639	R_1 = 0.0314, wR_2 = 0.0783
R indices (all data)	R_1 = 0.0419, wR_2 = 0.1007	R_1 = 0.0259, wR_2 = 0.0645	R_1 = 0.0369, wR_2 = 0.0822
Largest diff. peak/hole (e·Å^{-3})	0.755 and –0.476	0.169 and –0.313	0.328 and –0.414
CSD no.	2170399	2170396	2170395

Table A2. Crystallographic details for complexes (**6a**) – (**7a**) using 3-NO_2-5-OMe-sal_2-323 as ligand.

Compound	(**6a**)	(**7a**)·MeOH·0.5MeCN	(**7a**)·2EtOH
X^-	PF_6^-	BPh_4^-	BPh_4^-
sample code	CCDC 2170398	CCDC 2170394	CCDC 2170393
Empirical formula	$C_{24}H_{30}N_6O_8F_6PMn$	$C_{100}H_{111}B_2N_{13}O_{18}Mn_2$	$C_{52}H_{62}BN_6O_{10}Mn$
Formula weight	730.45	1914.52	996.83
Temperature (K)	100(2)	100(2)	100(2)
Radiation	Cu-Kα	Mo-Kα	Cu-Kα
Crystal system	triclinic	monoclinic	monoclinic
Space group	P-1 (#2)	$P2_1/n$ (#14)	$P2_1/n$ (#14)
Crystal size (mm)	0.164 x 0.093 x 0.076	0.381 x 0.326 x 0.260	0.227 x 0.153 x 0.067
a (Å)	8.7165(2)	13.6738(2)	13.65046(9)
b (Å)	9.2528(2)	27.2100(3)	27.2859(1)
c (Å)	17.8863(3)	14.4057(2)	14.46265(9)
α (°)	104.516(1)	90	90
β (°)	100.075(2)	112.176(2)	112.2153(7)
γ (°)	93.947(2)	90	90
V (Å^3)	1365.34(5)	4963.37(11)	4986.96(5)
Z	2	2	4
d_{calc} (g cm^{-3})	1.777	1.281	1.328
μ (mm^{-1})	5.437	0.327	2.682
F(000)	748	2012	2104
Limiting indices	h=±10, k=±11, l=±22	h=±18, k=±37, l=±19	h=±17, k=±34, l=±18
Reflect. coll./ uniq.	51916/5672	74510/12703	75196/10434
R(int)	0.0275	0.0198	0.0211
Complete to Θ (%)	98.6	98.5	99.3
Data/restr./param.	5672 / 0 / 417	12703 / 0 / 579	10434 / 0 / 609
GooF on F^2	1.078	1.064	1.084
Final R indices [I > 2σ(I)][a]	R_1 = 0.0389, wR_2 = 0.1187	R_1 = 0.0442, wR_2 = 0.1250	R_1 = 0.0344, wR_2 = 0.0933

R indices (all data)	R_1 = 0.0402, wR_2 = 0.1196	R_1 = 0.0477, wR_2 = 0.1274	R_1 = 0.0353, wR_2 = 0.0939
Largest diff. peak/hole ($e \cdot Å^{-3}$)	1.074 and –0.419	1.109 and –0.449	0.423 and –0.452
CSD no.	2170398	2170394	2170393

Table B1. Crystallographic details for complexes (**1b**) – (**3b**) using 3-OMe-5-NO_2-sal$_2$-323 as ligand.

Compound	(**1b**)·0.5EtOH	(**2b**)·0.5MeCN	(**3b**)
X^-	ClO_4^-	BF_4^-	Cl^-
CCDC code	CCDC 2170391	CCDC 2170392	CCDC 2170397
Empirical formula	$C_{50}H_{66}N_{12}O_{25}Cl_2Mn_2$	$C_{50}H_{63}B_2N_{13}O_{16}F_8Mn_2$	$C_{24}H_{30}N_6O_8ClMn$
Formula weight	1415.93	1385.63	620.93
Temperature (K)	100(2)	100(2)	100(2)
Radiation	Mo-Kα	Cu-Kα	Mo-Kα
Crystal system	monoclinic	monoclinic	orthorhombic
Space group	C2/c (#14)	C2/c (#14)	Pba2 (#32)
Crystal size (mm)	0.50 x 0.40 x 0.10	0.377 x 0.257 x 0.219	0.121 x 0.112 x 0.0997
a (Å)	35.871(5)	35.8800(6)	20.2841(3)
b (Å)	13.538(2)	13.5881(2)	7.6859(2)
c (Å)	12.4951(18)	12.3326(2)	8.3354(2)
α (°)	90	90	90
β (°)	100.275(3)	100.096(2)	90
γ (°)	90	90	90
V ($Å^3$)	5970.7(15)	5919.54	1299.50(5)
Z	4	4	2
d_{calc} (g cm^{-3})	1.575	1.555	1.587
μ (mm^{-1})	0.607	0.531	0.673
F(000)	2936	2856	644
Limiting indices	h=±42, k=±15, l=±14	h=±48, k=±18, l=±16	h=±30, K=±11, l=±12
Reflect. coll./ uniq.	21549/5118	64579/7766	22091/4543
R(int)	0.0378	0.0320	0.0389
Complete to Θ (%)	99.5	99.2	99.6
Data/restr./param.	5118 / 0 / 435	7766 / 0 / 435	4543 / 1 / 184
GooF on F^2	1.061	1.040	1.069
Final R indices [$I > 2\sigma(I)$][a]	R_1 = 0.0415, wR_2 = 0.0976	R_1 = 0.0311, wR_2 = 0.0758	R_1 = 0.0309, wR_2 = 0.0715
R indices (all data)	R_1 = 0.0563, wR_2 = 0.1059	R_1 = 0.0372, wR_2 = 0.0802	R_1 = 0.0382, wR_2 = 0.0757
Largest diff. peak/hole ($e \cdot Å^{-3}$)	0.523 and –0.302	0.393 and –0.445	0.324 and –0.359
CSD no.	2170391	2170392	2170397

Table B2. Crystallographic details for complexes (**4b**) – (**5b**) using 3-OMe-5-NO_2-sal_2-323 as ligand.

Compound	(**4b**)	(**4b**) (RT)	(**5b**)·0.5H_2O
X^-	Br^-	Br^-	OTf^-
CCDC code	CCDC 2170400	CCDC 2170401	CCDC 2170404
Empirical formula	$C_{24}H_{30}N_6O_8MnBr$	$C_{24}H_{30}N_6O_8MnBr$	$C_{50}H_{62}N_{12}O_{23}F_6S_2Mn_2$
Formula weight	665.39	665.39	1487.11
Temperature (K)	100(2)	293(2)	100(2)
Radiation	Mo-Kα	Mo-Kα	Cu-Kα
Crystal system	orthorhombic	orthorhombic	monoclinic
Space group	Pba2 (#32)	Pba2 (#32)	$P2_1/c$ (#14)
Crystal size (mm)	0.421 x 0.286 x 0.163	0.429 x 0.294 x 0.176	0.340 x 0.213 x 0.169
a (Å)	20.1943(5)	20.3147(4)	27.2827(2)
b (Å)	7.9080(2)	7.9972(2)	10.14482(5)
c (Å)	8.3110(2)	8.3422(2)	22.3215(1)
α (°)	90	90	90
β (°)	90	90	96.6945(5)
γ (°)	90	90	90
V ($Å^3$)	1327.24(6)	1355.28(5)	6135.98(6)
Z	2	2	4
d_{calc} (g cm^{-3})	1.665	1.631	1.610
μ (mm^{-1})	2.063	2.021	4.937
F(000)	680	680	3064
Limiting indices	h=±30, k=±11, l=±12	h=±30, k=±12, l=±12	h=±34, K=±12, l=±28
Reflect. coll./ uniq.	24353/4665	20348/4687	97859/12914
R(int)	0.0330	0.0247	0.0340
Complete to Θ (%)	99.8	99.8	100.0
Data/restr./param.	4665 / 1 / 184	4687 / 1 / 184	12914 / 0 / 863
GooF on F^2	1.057	1.056	1.046
Final R indices [I > 2σ(I)][a]	R_1 = 0.0294, wR_2 = 0.0665	R_1 = 0.0314, wR_2 = 0.0711	R_1 = 0.0328, wR_2 = 0.0867
R indices (all data)	R_1 = 0.0339, wR_2 = 0.0691	R_1 = 0.0405, wR_2 = 0.0757	R_1 = 0.0341, wR_2 = 0.0879
Largest diff. peak/hole (e·$Å^{-3}$)	0.612 and –0.448	0.397 and –0.404	0.854 and –0.675
CSD no.	2170400	2170401	2170404

Table B3. Crystallographic details for complexes (**6b**) – (**7b**) using 3-OMe-5-NO_2-sal_2-323 as ligand.

Compound	(**6b**)	(**6b**) (180 K)	(**7b**)
X^-	NTf_2^-	NTf_2^-	BPh_4^-
CCDC code	CCDC 2170403	CCDC 2170405	CCDC 2170402
Empirical formula	$C_{26}H_{30}N_7O_{12}F_6S_2Mn$	$C_{26}H_{30}N_7O_{12}F_6S_2Mn$	$C_{48}H_{50}BN_6O_8Mn$

Formula weight	865.63	865.63	904.69
Temperature (K)	100 (2)	180(2)	100(2)
Radiation	Cu-Kα	Cu-Kα	Cu-Kα
Crystal system	triclinic	triclinic	monoclinic
Space group	P-1 (#2)	P-1 (#2)	$P2_1/c$ (#14)
Crystal size (mm)	0.281 x 0.231 x 0.074	0.158 x 0.111 x 0.076	0.268 x 0.226 x 0.157
a (Å)	13.94843(9)	14.08625(8)	12.09031(9)
b (Å)	15.0415(1)	15.18827(9)	19.8920(2)
c (Å)	17.7750(1)	17.79994(9)	18.8961(2)
α (°)	95.7942(5)	95.4094(4)	90
β (°)	110.5680(5)	110.5229(5)	99.4532(6)
γ (°)	96.4949(5)	98.0812(4)	90
V ($Å^3$)	3429.25(4)	3488.53(4)	4482.81(7)
Z	4	4	4
d_{calc} (g cm^{-3})	1.677	1.648	1.340
μ (mm^{-1})	5.219	5.130	2.896
F(000)	1768	1768	1896
Limiting indices	h=$\pm$17, k=$\pm$18, l=$\pm$22	h=$\pm$17, k=$\pm$19, l=$\pm$22	h=$\pm$15, K=$\pm$25, l=$\pm$23
Reflect. coll./ uniq.	135038/14338	139656/14552	49389/9401
R(int)	0.0455	0.0543	0.0280
Complete to Θ (%)	100.0	100.0	100.0
Data/restr./param.	14338 / 0 / 1020	14552 / 0 / 1020	9401 / 0 / 579
GooF on F^2	1.032	1.048	1.038
Final R indices [I > 2σ(I)][a]	R_1 = 0.0318, wR_2 = 0.0867	R_1 = 0.0332, wR_2 = 0.0824	R_1 = 0.0298, wR_2 = 0.0781
R indices (all data)	R_1 = 0.0331, wR_2 = 0.0880	R_1 = 0.0427, wR_2 = 0.0884	R_1 = 0.0319, wR_2 = 0.0798
Largest diff. peak/hole (e·$Å^{-3}$)	0.749 and –0.547	0.513 and –0.399	0.271 and –0.441
CSD no.	2170403	2170405	2170402
